# A scoping review and proposed workflow for multi-omic rare disease research

**DOI:** 10.1186/s13023-020-01376-x

**Published:** 2020-04-28

**Authors:** Katie Kerr, Helen McAneney, Laura J. Smyth, Caitlin Bailie, Shane McKee, Amy Jayne McKnight

**Affiliations:** 1 Centre for Public Health, Queen’s University Belfast, Belfast, Northern Ireland; 2 Regional Genetics Centre, Belfast City Hospital, Level A, Tower Block, Lisburn Road, Belfast, BT9 7AB Northern Ireland

**Keywords:** Epigenomics, Exomics, Genomics, Methylomics, Multi-omics, Rare disease, Transcriptomics, Whole exome sequencing, Whole genome sequencing

## Abstract

**Background:**

Patients with rare diseases face unique challenges in obtaining a diagnosis, appropriate medical care and access to support services. Whole genome and exome sequencing have increased identification of causal variants compared to single gene testing alone, with diagnostic rates of approximately 50% for inherited diseases, however integrated multi-omic analysis may further increase diagnostic yield. Additionally, multi-omic analysis can aid the explanation of genotypic and phenotypic heterogeneity, which may not be evident from single omic analyses.

**Main body:**

This scoping review took a systematic approach to comprehensively search the electronic databases MEDLINE, EMBASE, PubMed, Web of Science, Scopus, Google Scholar, and the grey literature databases OpenGrey / GreyLit for journal articles pertaining to multi-omics and rare disease, written in English and published prior to the 30th December 2018. Additionally, The Cancer Genome Atlas publications were searched for relevant studies and forward citation searching / screening of reference lists was performed to identify further eligible articles. Following title, abstract and full text screening, 66 articles were found to be eligible for inclusion in this review. Of these 42 (64%) were studies of multi-omics and rare cancer, two (3%) were studies of multi-omics and a pre-cancerous condition, and 22 (33.3%) were studies of non-cancerous rare diseases. The average age of participants (where known) across studies was 39.4 years. There has been a significant increase in the number of multi-omic studies in recent years, with 66.7% of included studies conducted since 2016 and 33% since 2018. Fourteen combinations of multi-omic analyses for rare disease research were returned spanning genomics, epigenomics, transcriptomics, proteomics, phenomics and metabolomics.

**Conclusions:**

This scoping review emphasises the value of multi-omic analysis for rare disease research in several ways compared to single omic analysis, ranging from the provision of a diagnosis, identification of prognostic biomarkers, distinct molecular subtypes (particularly for rare cancers), and identification of novel therapeutic targets. Moving forward there is a critical need for collaboration of multi-omic rare disease studies to increase the potential to generate robust outcomes and development of standardised biorepository collection and reporting structures for multi-omic studies.

## Background

The scale of the rare disease challenge is a staggering one, with upwards of 8000 types of rare diseases described and an estimated 262.9–446.2 million people living with a rare disease globally. The definition of a rare disease varies internationally; the European definition is any disease with an incidence of less than one in 2000 [1], the United States (US) definition is conditions affecting fewer than 200,000 people [2], and the Chinese definition is disorders prevalent in less than one in 500,000 within the population [3]. Yet while the type and definitions of a rare disease may vary, there are many common issues faced by patients falling under the ‘rare’ umbrella.

The first hurdle many patients’ face is escaping the ‘diagnostic odyssey’, with an average of 5.6 years waiting for an accurate diagnosis in the United Kingdom (UK) and 7.6 years in the US [4]. Patients often report receiving several inaccurate diagnoses before the correct conclusion is reached. Obtaining a diagnosis has a significant impact on the development of a patients' defined care pathway as an accurate diagnosis can enable appropriate medical intervention, access to public services (such as financial support) and connection with vital rare disease support groups [5–7]. The difficulties in providing a diagnosis arise due to several interacting factors. Rare diseases have been widely reported as presenting with phenotypic and genetic heterogeneity which can make them difficult to diagnose even by specialists with prior experience [8–11], and the often multi-system impact of the conditions can mean they are masked by common complex disease symptoms [12]. Overlapping phenotypes in patients with more than one rare disease can also be difficult to differentiate and provide a conclusive diagnosis [13]. As rare diseases frequently have multi-system impact, patients are usually managed by more than one physician across a range of medical specialities. For example, a national survey of rare disease patients and carers in Northern Ireland showed that 63% of participants reported attending multiple doctors with 7% reporting management by greater than 10 doctors [14]. The nature of typical patient confidentiality can make essential communication between healthcare teams difficult, particularly with care across multiple centres and when accessing external specialist centres of excellence, thus leading to further delays in the diagnosis of a rare disease and fragmented patient care [4, 15, 16].

Even where a patient is fortunate enough to obtain a diagnosis there are often limited or no treatment options available, and a third of all rare disease patients die before reaching their fifth birthday [2]. Conditions lasting into adulthood are often debilitating and/or life limiting. The development of a panel of sensitive, minimally invasive and clinically accessible molecular biomarkers for faster diagnosis of patients with rare diseases will facilitate optimised care strategies and drive new therapeutic developments. This will be aided by the evolution of international registries, federated datasets, and computational tools that enable secure sharing and analysis of complex data generated within rare disease research networks; one such example of evolving infrastructure for rare disease is the Health Data Research UK (HDRUK) SPRINT exemplar innovation program which, in collaboration with the National Institute for Health Research (NIHR) BioResource and several National Health Service (NHS) trusts, aims to provide a dedicated research resource involving integration of phenotype-genotype information through cloud-based methods [17].

The advent of high-throughput technology in the past decade, such as next generation sequencing (NGS) and high-density microarrays have enabled large scale genomic analysis of rare diseases and brought hope for many patients and their families [18]. For example, t*he 100,000 Genomes Project* was a UK based project which recently completed whole genome sequencing (WGS) of 119,286 genomes including those from 74,674 patients with rare diseases and their family members, providing actionable findings for 20–25% of rare disease cases where traditional genetic testing did not identify a causal variant; additionally, new therapeutic targets have been identified and this transformational research project that was embedded with the UK NHS is moving towards generating sequencing data for ~ 1 million individuals [19]. Thirty percent of the identified causal variants found by the 100,000 Genomes pilot project had not previously been reported [20]. Moving forward the Health and Social Care secretary announced in 2018 plans to continue this work by sequencing five million genomes in the UK over the next five years, with all seriously ill children being offered WGS from 2019 [21]. Rapid genome/exome sequencing for acutely ill children with a likely genetic diagnosis will enable improve diagnostic rates with rapidly implemented optimised care protocols. Non-invasive prenatal testing that analyses foetal cell free circulating DNA within a maternal blood sample to identify chromosomal disorders has been introduced by many countries, including tests such as Harmony (Ireland, UK, US, Spain, Mexico, Germany, Canada and more) and MaterniT21 (US, Algeria, Belgium, Cameroon, Czech Republic, France and more) [22]. International projects such as the US National Institute of Health (NIH) Undiagnosed Diseases Program (UDP) aims to provide a diagnosis and identify treatment options, whilst the International Rare Diseases Research Consortium (IRDiRC) aims to provide a diagnosis for rare disease patients within one year of presentation, to develop 1000 new therapies and assess the impact of these diagnoses and novel therapies by 2027 [23–25].

**Fig. 1:**
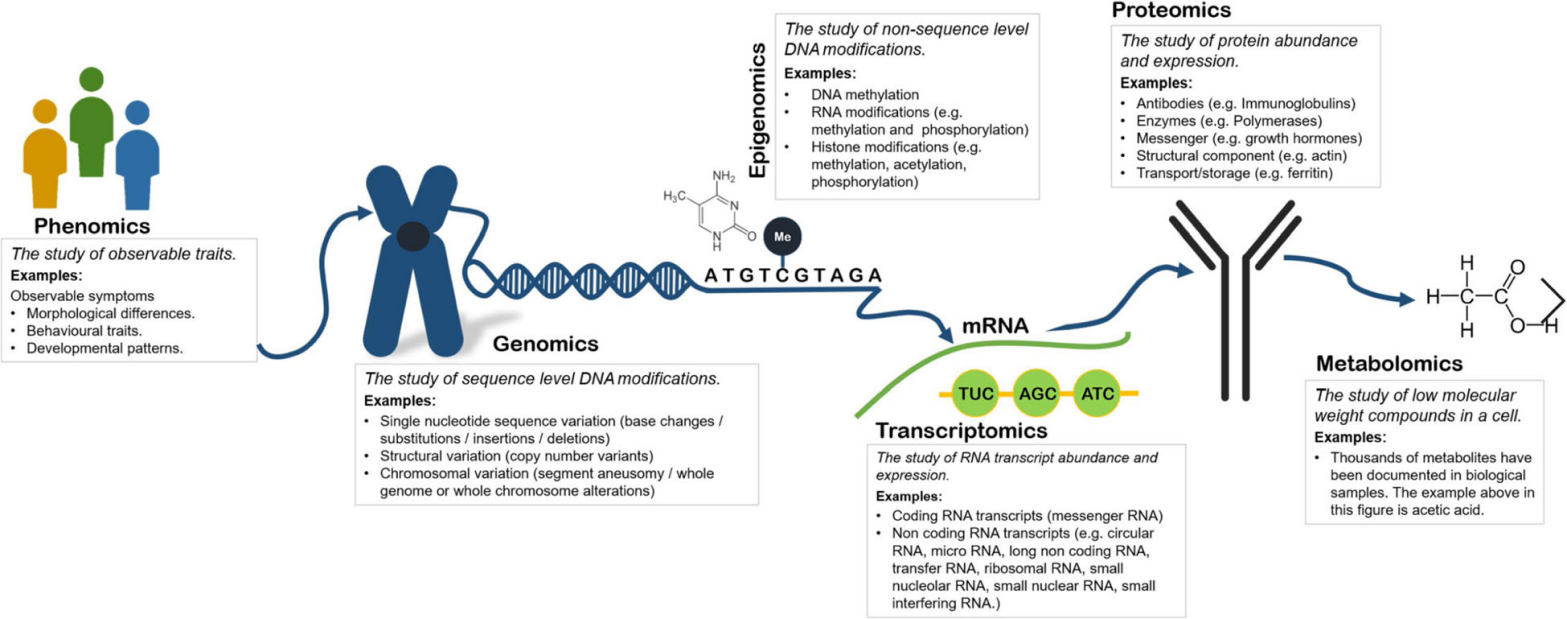
The diagram emphasises the potential of studies which, following careful phenotyping at study conception, utilise integrated multi-omic analysis to consider multiple components in the journey from DNA to expression

Undoubtedly WGS efforts have been found to increased diagnostic yield, with the figure ranging from 21 to 73% depending on participant age and phenotype [18]. However, for those phenotypes with lower diagnostic yields, WGS by projects such as *The 100,000 Genomes Project* facilitate further investigations by providing a platform for integrative multi-omic analysis. The term ‘omic’ stems from the suffix ‘ome’ added to many fields of biological study, which refers to the study of something in its entirety. There are estimated to be over 500 omic types [26], (Table S4) with the most commonly known being genomics, epigenomics, transcriptomics, proteomics, metabolomics and phenomics (definitions for common examples can be found in Fig. 1). Considered individually, these omic types have been used to identify and / or provide functional supporting information for candidate pathogenic mutations for rare diseases across various medical specialities. For example, transcriptomics from blood samples has been shown as a useful method of characterising undiagnosed rare diseases with a validated diagnostic yield of 7.5% where whole exome sequencing (WES) was insufficient to identify a causal variant [27]. Taking a holistic molecular approach by integrating analyses for several different ‘omic’ types could further increase diagnostic yield and contribute to understanding of phenotypic heterogeneity and disease progression. Furthermore, multi-omic analysis could illuminate opportunities for drug repurposing through identification of novel therapeutic targets, an important component of rare disease treatment, where drugs originally intended for treatment and management of common complex diseases can be applied for use in rare diseases where there is unlikely to be many existing treatment options [28]. In reality, the full potential of integrative multi-omic analysis has yet to be comprehended. Challenges exist in the integration and processing of large datasets across ‘omics’ technologies and even between laboratories (with much data now publicly available online), as well as interpreting the clinical impact of the relationships between these omic analyses [29].

### Aims and objectives

To fully understand what research has been undertaken and what gaps still exist, this scoping review aims to systematically summarise research into multi-omics and rare disease research by:

- Evaluating what primary research studies exist pertaining to multi-omics and rare disease and which type of omic analysis was undertaken.
- Highlighting research outcomes with implications for rare disease diagnosis, treatment or improved understanding of disease mechanisms.

## Main text

### Methods summary

The full methodology for this review is available online as a published protocol [30], and follows the Joanna Briggs Institute methodology guidance for scoping reviews. To ensure our search was comprehensive, we followed all applicable aspects of the Preferred Reporting Items for Systematic Reviews and Meta-Analyses Extension for Scoping Reviews guidelines [31].

With reference to the population, concept, context (PCC) guidelines for determining the review research question, our population of interest was studies of patients diagnosed with a rare disease, meeting the European definition (an incidence of less than 5 in 1000) [32], or with a rare cancer (European definition of less than 6 in 100,000 and the US definition of less than 15 in 100,000) [33, 34]. Our concept was multi-omic data generated on rare diseases, where a multi-omic study was defined as one which included two or more omic analyses types [26]. The context of the scoping review was primary studies written in English, published prior to 30th December 2018.

Databases searched included MEDLINE, EMBASE, PubMed, Web of Science, Scopus and Google Scholar, as well as the grey literature databases GreyLit and OpenGrey. One additional information source utilised not detailed in the published protocol, was papers published by The Cancer Genome Atlas (TCGA). This resource was identified through an article returned in the initial search. TCGA is a large collaborative project between the National Cancer Institute and the National Human Genome Research Institute, which has conducted multi-omic analyses of 33 cancers [35]. While no hard definition of a rare cancer was used by TCGA, researchers selected uncommon cancers on the basis of public health impact and the feasibility of getting enough samples for meaningful analyses. Review articles and reference lists were searched for any additional eligible articles, as well as forward citation searching using the Web of Science Cited Reference Search Tool. For any conference abstracts identified, full texts were searched.

The reference management software EndNote X8 was used for citation handling throughout duplicate removal and title/abstract screening. Microsoft Excel was used to record results and exclusion reasons, as well as for full text screening and data extraction. Data extraction (otherwise referred to in scoping reviews as data-charting) was performed independently and in duplicate (by KK and CB) with any discrepancies were resolved by consultation of a third individual. Data extracted included rare diagnosis (or phenotype where patients were undiagnosed), omic analyses type, study design information, experimental methods and key relevant results. As is typical of scoping reviews, a qualitative narrative synthesis was then conducted to summarise key components of the multi-omic rare disease field [31, 36, 37].

## Results

**Fig. 2:**
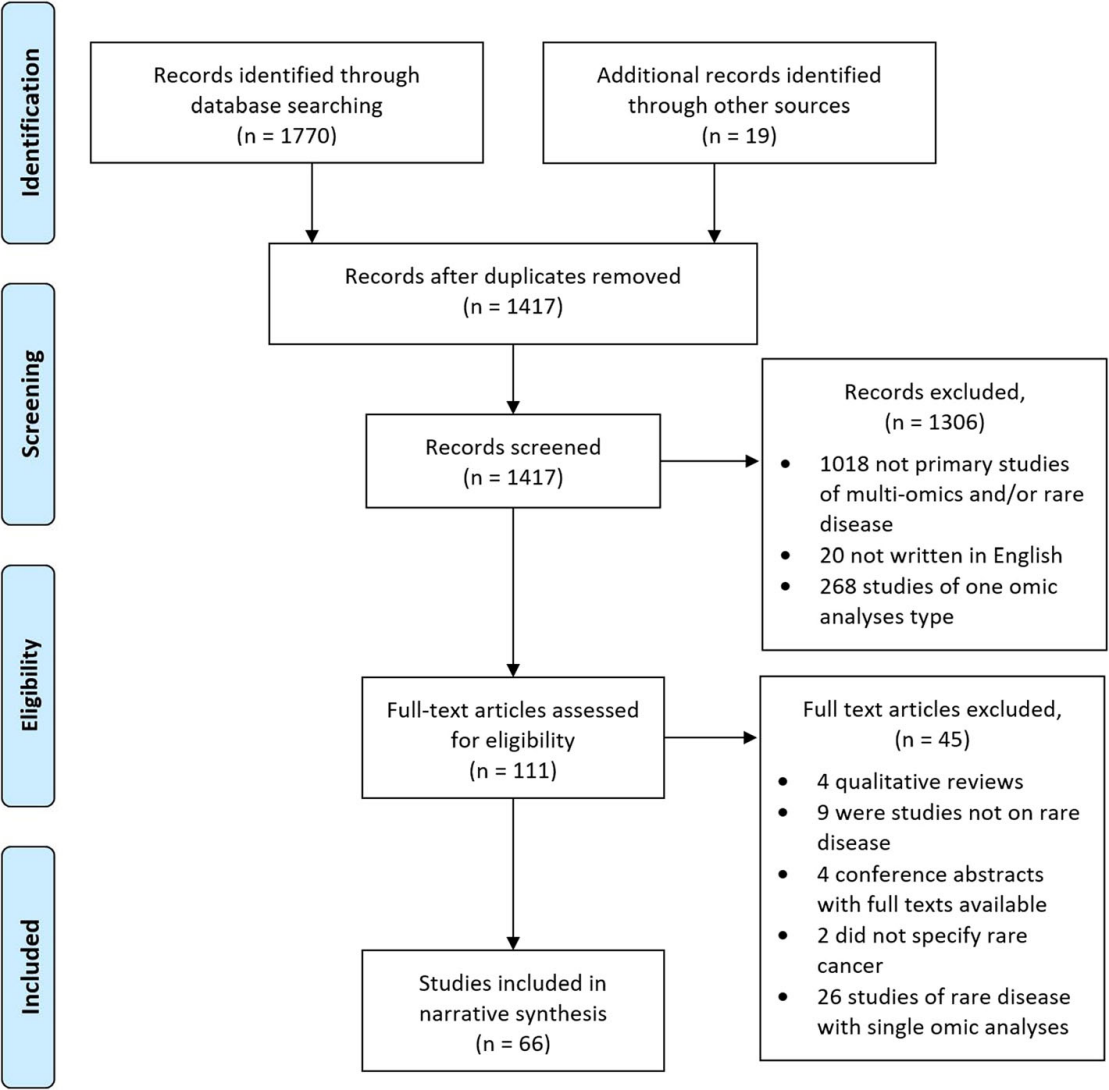
PRISMA flow diagram summarising the screening process. 66 articles were selected for final inclusion in the review [31].

**Fig. 3:**
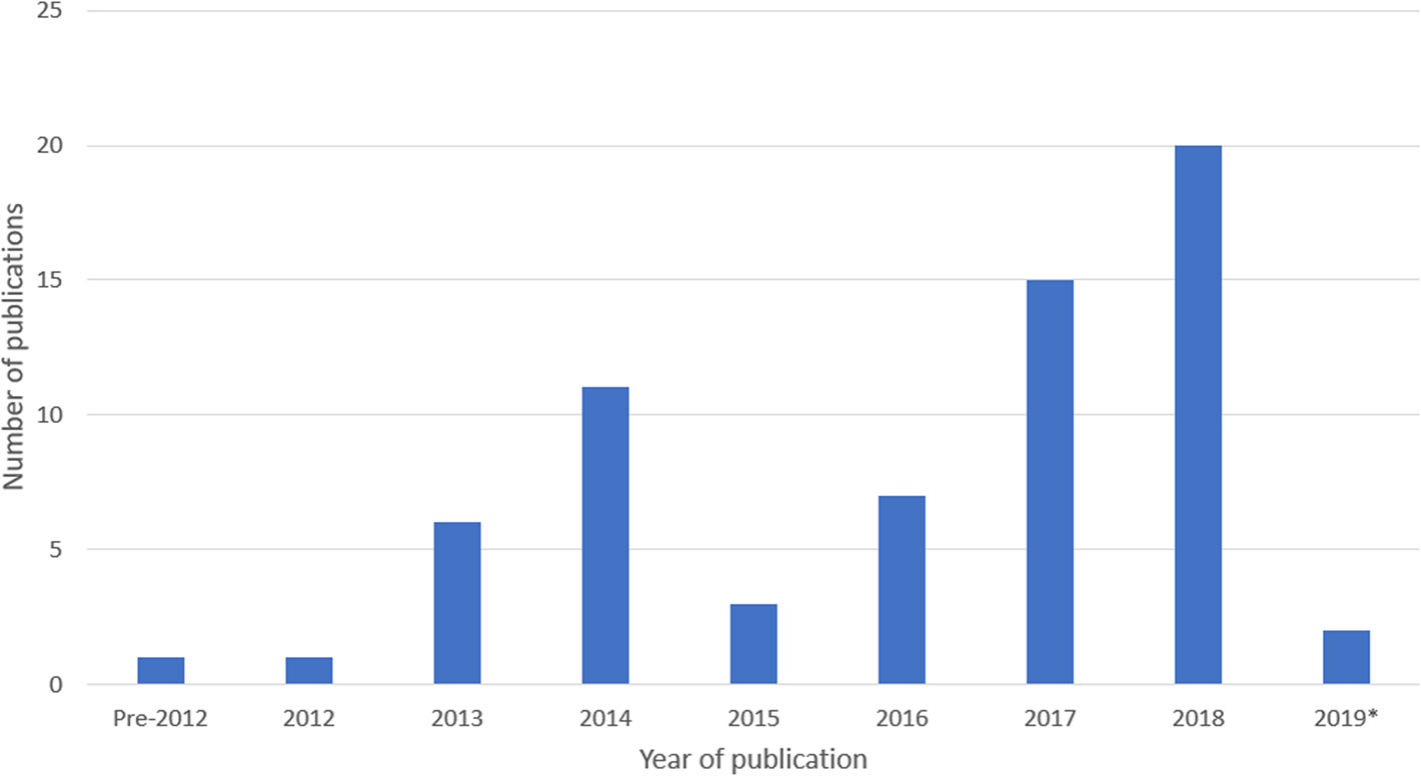
A significant increase is seen the publication of multi-omic studies of rare disease between since 2012, with 22 studies conducted since 2018 (33%), *2019 representing a partial year outside of the original date restrictions as these articles were returned in the additional search of TCGA publications (see methods summary)

Initial searches identified a total of 1770 articles: *n* = 173 MEDLINE articles, *n* = 630 EMBASE articles, n = 17 PubMed articles, *n* = 206 Google Scholar articles, *n* = 721 Web of Science articles, *n* = 23 Scopus articles. A further 19 articles were identified from additional sources, not included in the initial search numbers. This included five articles which were full text versions of conference abstracts [38–42]. One paper returned in the initial search published through TCGA [43], led to the identification of a further 13 articles on multi-omics of rare cancers [44–56]. Finally, one article was identified from the reference list of a review paper [57, 58]. The screening process is summarised in Fig. 2. Following duplicate removal, 1417 articles were identified for title/abstract screening from which 1306 articles were excluded (1018 papers as they were not primary studies of multi-omics and/or rare disease, 20 articles as they were not written in English, and 268 articles as they only included one omic analysis type). This left 111 articles for full text screening, from which four articles were excluded as they were qualitative review articles, four as they were conference abstracts and the corresponding full texts were already included in the return, nine articles as they were found not to be primary studies of rare disease, two did not specify which rare cancer and finally a further 26 articles described only a single omic type (a total of 45 articles removed at this stage). Subsequently, 66 articles were eligible for inclusion in this review. General study and participant characteristics are summarised in Table 1, detailed experimental procedures and results are available in Additional file 1: supplementary Table 1 (Table S1). The year of publication ranged from 2001 to 2018, with a rapid increase in publications over the past decade (Fig. 3). Two of the final 66 included papers were published in 2019, despite the 2018 date restriction, as these were identified from within the additional TCGA search [38, 59]. Evolution of inclusion and exclusion criteria is not unusual within the process of conducting scoping reviews [37]. Three study designs were identified: case-control studies (*n* = 55), familial studies (*n* = 6) and studies which incorporated a mix of both familial comparisons and external unrelated cohort comparisons (n = 5) into their methodological designs (Table 1).

**Table 1:** Summary of general study characteristics

Study Characteristic	Number of Studies (***n*** = 66)	Percentage
*Publication year*		
2000–2010	1	1.52%
2011–2015	21	31.82%
2016–2019	44	66.67%
*Study design*		
Case-control	55	83.33%
Familial study	6	9.09%
Case-control and familial study	5	7.58%
*Number of participants in studies*		
1–5	21	31.82%
6–10	3	4.55%
11–20	6	9.09%
21–50	5	7.58%
51–100	12	18.18%
101–200	7	10.61%
201–500	5	7.58%
> 1000	2	3.03%
Not applicable (animal models)	3	4.55%
Unknown	2	3.03%
*Participant age*		
0–10 years	10	15.15%
11–20 years	1	1.52%
21–30 years	2	3.03%
31–40 years	1	1.52%
41–50 years	6	9.09%
51–60 years	8	12.12%
61–70 years	9	13.64%
Not applicable (animal models)	3	4.55%
Unknown	26	39.39%
*Participant race/ethnicity*	*Total known: 1534 participants*	
Arab	10 participants	0.65%
Asian	150 participants	9.78%
Black/African/African-American	100 participants	6.52%
Caucasian	1259 participants	82.07%
Hispanic/Latino	10 participants	0.65%
Mixed	5 participants	0.33%
Not applicable	3 studies	–
Unknown	40 studies	–

Four of the 66 articles were conference abstracts for which no full text was available, but appeared to describe case-control studies. As expected, likely due to the low prevalence of rare diseases, no randomised controlled trials were identified in the search. The most frequent number of participants was 1–5, with mode reported instead of mean as there are a number of studies conducted by TCGA which have extremely large participant numbers which would disproportionally skew the mean of included studies. The mean age of participants was 39.4 years (median = 49), however this was not reported for almost half of the included articles (39.4%, 26 studies). There was a peak in the number of studies which included participants between 0 and 10 years of age, followed by a significant reduction until a second peak from 50 years of age (Table 1). Similar to participant age, participant ethnicity/race was unknown in a large percentage of included studies (60.6%, 40 studies). Where ethnicity and/or race were known there was significant heterogeneity in reporting, therefore these have been summarised in groups in Table 1. The most common participant ethnicity was Caucasian (82.1%), and the least common ethnicity mixed race (0.33%). Publication countries of origin included the United States of America (*n* = 38), France (*n* = 5), Switzerland (*n* = 4), the United Kingdom (n = 4), Canada (n = 3), Japan (n = 3), Germany (n = 3), Italy (*n* = 2) Brazil (*n* = 1), Finland (n = 1), Korea (n = 1), Spain (n = 1).

**Table 2:** Fourteen combinations of omic analyses for rare disease research

‘Omic’ analyses combination	Number of Studies (***n*** = 66)	Percentage
Epigenomics, genomics	1	1.52%
Epigenomics, genomics, proteomics, transcriptomics (TCGA)	13	19.70%
Epigenomics, genomics, transcriptomics	9	13.64%
Epigenomics, proteomics, transcriptomics	2	3.03%
Epigenomics, transcriptomics	1	1.52%
Genomics, metabolomics	4	6.06%
Genomics, metabolomics, phenomics	1	1.52%
Genomics, phenomics	1	1.52%
Genomics, phenomics, transcriptomics	2	3.03%
Genomics, proteomics	7	10.61%
Genomics, proteomics, transcriptomics	8	12.12%
Genomics, transcriptomics	13	19.70%
Metabolomics, proteomics	1	1.52%
Proteomics, transcriptomics	3	4.55%

**Fig. 4:**
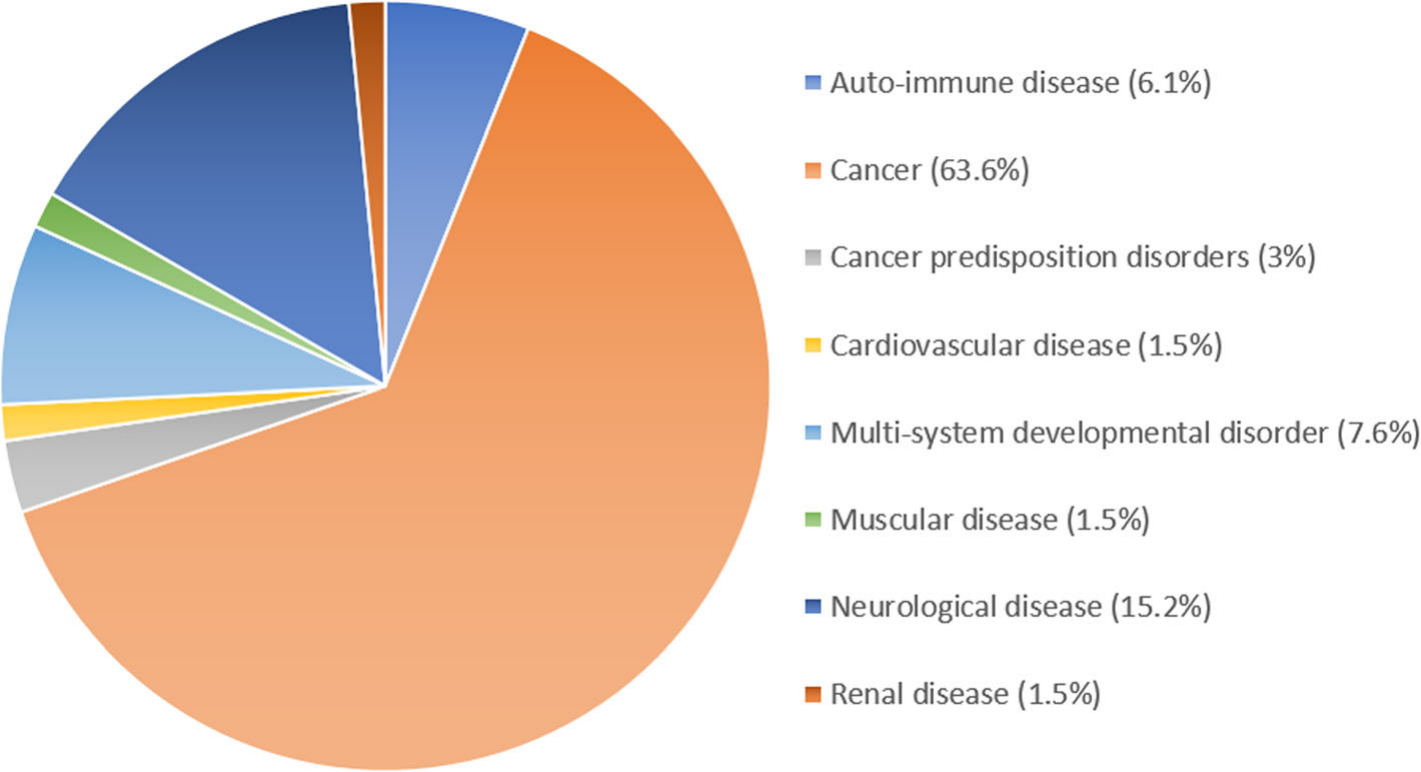
Multi-omic studies of rare disease are primarily conducted on rare cancers (64%, 42 studies). Two studies of pre-cancerous disorders were included (3%), and the remaining 22 studies (33%) were of non-cancerous rare diseases

Fourteen different omic analyses types were identified within this scoping review, including various combinations of genomic, epigenomic, metabolomic, phenomic, proteomic and transcriptomic analyses (Table 2), with transcriptomics being the most commonly integrated omic analyses type. The majority of studies eligible for inclusion were rare cancers (64%, 42 studies), including two studies of pre-cancerous conditions, summarised in Table 3. Of the remaining 22 non-cancerous rare disease articles, neurological disorders was the most common disease type (15%, 10 studies) whilst other rare disease types combined contributed just 20% of the included studies. These included auto-immune diseases, multi-system developmental disorders, cardiovascular disease, muscular disease, neurological disease and renal disease. Specific rare diseases are detailed in the discussion and in Additional file 1, Table S1. Studies of rare cancers/pre-cancerous rare diseases had more than ten times the mean participant number compared to studies of non-cancerous rare diseases (429.3 ± 1799.5 and 41.2 ± 113.6 mean and standard deviation of participant numbers respectively). However, this was influenced by two studies with high participant numbers (3527 and 11,286 participants) [43, 60]. The disproportionate representation of cancerous to non-cancerous rare diseases is summarised in Fig. 4.

**Table 3:** Summary of participant diagnosis/phenotype and rare cancer types

Study Characteristic	Number (***n*** = 66)	Percentage
*Rare disease, cancer or phenotype*		
Acute myeloid leukaemia	1	1.52%
Adrenocortical carcinoma	5	7.58%
Autoinflammatory disorder	1	1.52%
Brain cancer	4	6.06%
Cancer predisposition disorder	2	3.03%
Cardiovascular disorder	1	1.52%
Cholangiocarcinoma	1	1.52%
Chromosomal disorder	1	1.52%
Fibrolamellar hepatocellular carcinoma	1	1.52%
Gastric cancer	2	3.03%
Gynaecological cancer	4	6.06%
Immune Disorder	3	4.55%
Malignant pleural mesothelioma	1	1.52%
Metabolic disorder	1	1.52%
Mixed rare cancers (TCGA)	2	3.03%
Multi-system developmental disorder	3	4.55%
Muscular disorder	1	1.52%
Neurological disorder	7	10.61%
Neurometabolic disorder	2	3.03%
Neuromuscular disorder	1	1.52%
Pheochromocytomas and paragangliomas	1	1.52%
Phyllodes breast tumours	1	1.52%
Primary testicular germ cell tumours	1	1.52%
Primary urethral clear-cell adenocarcinoma	1	1.52%
Prostate cancer	2	3.03%
Pseudomyxoma peritonei	1	1.52%
Rare renal cancer	2	3.03%
Renal disorder	1	1.52%
Salivary duct carcinoma	1	1.52%
Sarcoma	5	7.58%
Sezary tumour	1	1.52%
Thymic epithelial cancer	2	3.03%
Thyroid cancer	2	3.03%
Uveal Melanoma	1	1.52%

From the data extraction process five key themes were identified in this review which are expanded upon in a narrative synthesis in the discussion section. These included:

1. Significant use of NGS technologies and high throughput microarrays for multi-omic rare disease analysis.
2. Varied methodological and analytical approaches to multi-omic rare disease research.
3. Multi-omics for diagnosis of undiagnosed rare phenotypes.
4. Multi-omics for identification of pathogenic and prognostic biomarkers of rare disease.
5. Multi-omics for elucidation of novel treatments and drug re-purposing opportunities.

A concise critical appraisal of studies was conducted using a checklist adapted from the Joanna Briggs Institute (JBI) critical appraisal tools in the PRISMA extension for scoping reviews (Additional file 2: Table S2) [31]. Conference abstracts were excluded from critical appraisal (*n* = 5). Assessment of sample numbers, appropriate matching of cases and controls (e.g. age/gender), appropriate experimental controls and statistical analysis (e.g. accounting for multiple variates) lead to the identification of 19 studies with high methodological rigour, 10 with medium methodological rigour and 32 with low methodological rigour (Additional file 1: Table S1). This high proportion of studies deemed to have low methodological rigour was in most cases due to very low sample numbers, e.g. case reports of one person, and is typical of studies of rare disease.

## Discussion

Scoping reviews are an increasingly popular method of summarising literature in a researcher’s particular area of interest, which can be used to identify themes and significant gaps to inform research hypothesis development [61]. This scoping review provides a comprehensive narrative synthesis of studies of multi-omics and rare disease [36], identifying 66 primary studies published between 2000 and 2019. Estimated European prevalence (where known) of each rare disease and overall study objectives are summarised in Table 4, whilst detailed study design, methodology and results are available from Additional file 1, Table S1.

**Table 4:** Rare disease prevalence in Europe, reference numbers of relevant studies and key objectives of these research papers

Rare disease	Estimated prevalence	Overall study objectives and reference number(s)
*Rare cancers*		
Acute myeloid leukaemia	5–8/100,000 [62]	• Molecular characterisation of cancers by TCGA^a^ across tissues of origin [43, 60]. • Identification of pathogenic genomic and epigenomic variants [44].
Adrenocortical carcinoma	0.7–2/1 million [63]	• Identification of pathogenic genomic, epigenomic, proteomic and transcriptomic variants [41, 64]. • Identification of prognostic genomic, epigenomic and transcriptomic biomarkers [65]. • Molecular characterisation of cancers by TCGA across tissues of origin [39, 43]. • Identification of novel therapeutic targets through genomics, transcriptomics and proteomics [66].
Central nervous system cancers	7/100,000 [67]	• Identification of pathogenic genomic and epigenomic variants [68]. • Molecular characterisation of ENB^b^ [69], ,R-GBM^c^ [70], and IGCTs^d^ [71]. • Molecular characterisation of cancers by TCGA across tissues of origin [43, 60].
Cholangiocarcinoma (Bile duct)	2.17/100,000 [72]	• Molecular characterisation of cancers by TCGA across tissues of origin [43, 45].
Diffuse large B-cell lymphoma	3.8/100,000 [73]	• Molecular characterisation of cancers by TCGA across tissues of origin [43].
Rare liver cancer (FL-HCC^e^)	1 in 5 million [74]	• Identification of pathogenic genomic and transcriptomic variants [75].
Gastric cancer	2.6/100,000	• Identification of pathogenic genomic, transcriptomic and epigenomic variants [76, 77].
Gynaecological cancer	USC^f^/UCS^g^: 2.57–5/100,000 [78, 79] SCCOHT^h^: 300 reported cases VSCC^i^: 2.5/100,000 [80]	• Molecular characterisation of cancers by TCGA across tissues of origin (USC/UCS) [43, 52, 53]. • Identification of novel therapeutic targets in SCCOHT through functional multi-omic analysis [42]. • Identification of pathogenic genomic and transcriptomic variants [81].
Mesothelioma	0.6–8/100,000 [82]	• Molecular characterisation of cancers by TCGA across tissues of origin [43, 46].
Oesophageal cancer	4.2/100,000 [83]	• Molecular characterisation of cancers by TCGA across tissues of origin [43].
Adrenal nerve tissue (PCCs^j^ and PGLs^k^)	0.4–0.21/100,000 [84]	• Molecular characterisation of cancers by TCGA across tissues of origin [48].
Phyllodes breast tumour	2.1/1 million [85]	• Identification of novel therapeutic targets through multi-omic analysis [86].
Rare urethral cancer (PUCA^l^)	0.31/100,000 [87]	• Molecular characterisation using cytopathology, genomics and transcriptomics [88].
Pseudomyxoma peritonei	1/1 million [89]	• Identification of prognostic biomarkers through genomics and proteomics [90].
Rare prostate cancers (SCPC^m^, CRPC-NE^n^)	Unknown prevalence.	• Molecular characterisation of SCPC using genomics and transcriptomics [91]. • Functional study which developed organoids to assess the molecular profile of CRPC-NE [92].
Rare renal cancers (ChRCC^o^, TLFRCC^p^)	Unknown prevalence.	• Molecular characterisation of cancers by TCGA across tissues of origin [43, 55, 93].
Salivary duct carcinoma	0.05–2/100,000 [94]	• Molecular characterisation of salivary duct carcinoma using proteomics and genomics [95]
Sarcoma	0.1–5/100,000 [96]	• Molecular characterisation of cancers by TCGA across tissues of origin [43, 49] • Identification of novel therapeutic targets through multi-omic analysis [97]. • Identification of pathogenic genomic and transcriptomic variants in angiosarcoma [98]. • Identification of prognostic multi-omic biomarkers [99].
Sézary syndrome	0.1/100,000 [100]	• Identification of novel therapeutic targets through genomic and transcriptomic analysis [101].
Testicular germ cell tumours	3.8–6.3/100,000 [102]	• Molecular characterisation of cancers by TCGA across tissues of origin [43, 50].
Thymoma and thymic cancers	1.3–3.2/1 million [103]	• Molecular characterisation of cancers by TCGA across tissues of origin [43, 51]. • Molecular characterisation and comparison between Asian/European thymic cancer profiles [104].
Thyroid cancer	2–6/100,000 [105]	• Molecular characterisation of cancers by TCGA across tissues of origin [43, 56]. • Identification of pathogenic epigenomic markers of medullary thyroid cancer development [106].
Uveal melanoma	5.1/1 million [107]	• Molecular characterisation of cancers by TCGA across tissues of origin [43, 54].
*Benign or pre-cancerous rare tumours*		
Rare head and neck cancer (MNTI^q^)	Unknown prevalence	• Identification of novel therapeutic targets using genomic and transcriptomic analysis [108].
Juvenile polyposis syndrome	1/100,000 [109]	• Molecular characterisation of the genomic, transcriptomic and proteomic profile [110].
*Non-Cancerous rare diseases*		
Mevalonate kinase deficiency	Unknown	• To explain polarised phenotypic heterogeneity in siblings with the same pathogenic mutation [111].
Triglyceride deposit cardiomyovasculopathy	Unknown	• Identification of pathogenic transcriptomic and proteomic markers of disease [112].
Monosomy 18p	1 / 50,0000 live births [113]	• Investigation of the role of monosomy 18p on FSHD^r^ type 2 development [114].
Rare auto-immune conditions	ICF1^r^ and IPEX^s^: 1/100,000 [115] PID^t^: 6/100,000 [116]	• Identification of pathogenic genomic, transcriptomic and epigenomic variants [117–119].
Congenital Disorder of Glycosylation	< 100 cases reported of each type [120]	• Investigation of key genomic and proteomic variants associated with glycosylation disorders [121].
Multi-system developmental disorders	TBS^u^: 1–9/100,000 [122] Primrose syndrome: 1/100,000 [123]	• Diagnosis of previously undiagnosed rare phenotypes [124]. • Identification of pathogenic genomic and proteomic variants [125].
Congenital absence of the ACL^v^/PCL^w^	1.7/100,000 live births	• Investigation of key genomic and proteomic variants associated with congenital ACL/PCL [126].
Rare neurological disease	SNS^x^ and Alexander’s disease: unknown Aconitase deficiency: 1/100,000 [127] HPE^y^: 1.31/100,0000 live births [128] Huntington’s: 7.2/million [129].	• Identification of genomic, proteomic, transcriptomic and metabolomic mutations [38, 130–132]. • Diagnosis of mitochondrial aconitase deficiency [58]. • Investigation of therapeutic intervention in animal models of Huntington’s disease [133].
Rare neuro-metabolic disease	Unknown, undiagnosed phenotypes.	• Diagnosis provision using phenomics, genomics and metabolomics [134, 135].
Rare neuro-muscular disease	Unknown, undiagnosed phenotypes.	• Diagnosis provision using genomics, transcriptomics and proteomics [136].
Rare renal disease (PUV^z^)	1/5000–8000 births [137]	• Prediction of post-natal prognosis in patients using peptidomics and metabolomics [138].

Abbreviations: *TCGA*^a^ The Cancer Genome Atlas, *ENB*^b^ Esthesioneuroblastoma, *R-GBM*^c^ Rhabdoid glioblastoma, *IGCTs*^d^ Intracranial germ cell tumours, *FL-HCC*^e^ Fibrolamellar hepatocellular carcinoma, *USC*^f^ Uterine serous carcinoma, *UCS*^g^ uterine carcinosarcoma, *SCCOHT*^h^ Small cell carcinoma of the ovary hypercalcemic type, *VSCC*^i^ Vulvar squamous cell carcinoma, *PCCs*^j^ Pheochromocytomas, *PGLs*^k^ paragangliomas, *PUCA*^l^ Primary Urethral Clear-Cell Adenocarcinoma, *SPPC*^m^ Small cell prostate cancer, *CRPC-NE*^n^ Castration resistant neuroendocrine prostate cancer, *ChRCC*^o^ Chromophobe renal cell carcinoma, *TLFRCC*^p^ Thyroid-like follicular renal cell carcinoma, *MNTI*^q^ Melanotic neuroectodermal tumour of infancy, *FSHD*^r^ Facioscapulohumeral muscular dystrophy, *ICF1*^*r*^ Immunodeficiency Centromere instability and Facial anomlies syndrome, *IPEX*^s^ Immune dysregulation polyendocrinopathy enteropathy X-linked, *PID*^t^ Primary immunodeficiency disorder, *TBS*^u^ Townes-Brocks syndrome, *ACL*^v^/*PCL*^w^ anterior/posterior cruciate ligaments, *SNS*^x^ Snyder-Robinson syndrome, *HPE*^y^ Holoprosencephaly, *PUV*^z^ Posterior urethral valves

Whilst this review conducted a comprehensive search, using multiple information sources and developing search terms carefully in collaboration with a Medical Faculty librarian, it was not possible to include all relevant studies, primarily due to the heterogeneity of terms used to identify these studies as multi-omics, and in varying definitions of a rare cancer. Rather it is intended that this scoping review will provide an overview of general themes in multi-omic rare disease research and provide direction for future projects. This is particularly true of multi-omics and cancer studies, which are conducted far more routinely than studies of non-cancerous rare diseases. Two studies of non-cancerous rare diseases which were eligible for inclusion, but not returned through our original search, were identified during peer-evaluation of this scoping review, both of which utilised RNA sequencing to increase diagnostic yield of Mendelian disorders reporting a 10% diagnosis in mitochondriopathy patients and 35% in undiagnosed rare muscular diseases respectively [139, 140]. A second limitation is that due to language restrictions we were only able to include articles written in English, which led to the exclusion of 20 articles. However, these articles are available in Additional file 2 (Table S3) and can be reviewed for readers able to interpret them. Following our published protocol, the search strategy can be easily reproduced by researchers hoping to conduct a multi-lingual inclusive search [30]. Furthermore, the vast majority (82%) of participants in the included studies for which ethnicity/race was known were identified as Caucasian, with all other ethnicities comprising just 17.9% of participants. It should be noted however that representation bias may have been introduced by the language limitations imposed on this review. Twenty articles were identified that were published in additional non-English languages; many of these may not meet the criteria for inclusion within the review and so the effect of such bias is likely to be minimal. This disproportionate representation of Western ethnicity will need to be addressed by international collaborative efforts in future research studies. In the narrative synthesis below, this review reflects how multi-omic rare disease research is the natural next step for progressing our understanding of rare diseases: whether that be for diagnostic or prognostic purposes, development of novel treatment options, or simply understanding the mechanisms behind disease progression. We also discuss the challenges posed by researchers attempting to conduct these projects and areas to be addressed in future projects.

### NGS, high density arrays and data integration software has enabled multi-omic research

The success of large-scale genomic analysis projects is largely owed to the development of cost-effective high throughput microarrays with semi-automated analysis and the refinement of NGS technologies. Similarly, emerging data for epigenomic and transcriptomic data typically use these approaches. Within the papers discussed in this review, platforms provided by Illumina® dominated for WGS, WES and RNA-seq data generation.

These included the:

- Illumina® Genome Analyzer_IIx_ System, developed 2008 (now obsolesced).
- Illumina® HiSeq™ 2000 and 2500, developed 2010 and 2012 respectively (now obsolesced).
- Illumina® MiSeq™, developed 2011.
- Illumina® HiSeqX series and NextSeq500®, developed 2014 (now discontinued).

More recent versions of Illumina technologies not used in this review include the NextSeq550® and the NextSeq 2000. These platforms, whilst undoubtedly very useful, rely on short read sequencing methods where the DNA is fragmented for sequencing and re-aligned against a reference genome for interpretation. Other providers of NGS less frequently seen included Ion Proton™ System by Ion Torrent™ and the Applied Biosystems™ 5500xl Genetic Analyzer. Moving forward with multi-omic rare disease research, long read sequencing methodologies (currently commercially provided by Oxford Nanopore Technologies and Pacific Biosciences) offer significant benefits compared to short read sequencing, with Oxford Nanopore also providing ultra-long read sequencing with additional benefits for identifying molecular variation. True long read sequencing has potential to overcome issues with amplification bias during short read library preparation (presuming the sample to be processed by LRS has not already underwent amplification), errors when aligning to a reference genome to due repetitive regions, detection of large structural or copy number variants and issues with inaccuracies in reference genomes themselves [141–143]. Furthermore, long read sequencing enables direct measurement of methylation and RNA sequencing without the need for reverse transcription to complementary DNA (cDNA) which can introduce additional errors [144, 145].

Other platforms utilised included microarrays for the detection of single nucleotide polymorphisms (SNP) such as the Affymetrix™ Genome-Wide Human SNP Array 6.0 (Applied Biosystems™), which enables the interrogation of approximately 900,000 SNPs across the genome. For studies which included epigenomic analysis of DNA methylation, the primary microarray platforms utilised were the Illumina® Infinium methylation arrays: the HumanMethylation27 (27 K), HumanMethylation450 and Illumina’s most recent array, the MethylationEPIC (850 K) BeadChip®. These arrays were used by all but two studies for DNA methylation, where one utilised targeted bisulphite pyrosequencing of four genes with the Qiagen PyroMark Q96 MD System [65], and a second utilised enhanced reduced representation bisulphite sequencing [92]. Proteomic and metabolomic analyses largely utilised liquid Chromatography with tandem mass spectrometry (LC-MS/MS) and nanoLC-MS/MS.

### A standardised methodological approach to multi-omic rare disease research is needed

One challenge with multi-omic rare disease research is the large variety of methodological approaches which researchers can choose to undertake. These can complicate data analysis due to between-laboratory batch effects and a lack of independent datasets generated from the same methods which may be needed to validate potential variants of interest. Therefore it would be valuable to develop a multi-omic analysis pipe-line that can be utilised to maximise the power of rare disease studies. As discussed previously, TCGA is a multi-centre cancer genomics programme run by the National Cancer Institute and National Human Genome Research Institute, which began in 2006 and has undertaken extensive multi-omic analysis of 33 cancers including several rare cancers [35]. Sixteen of the 66 articles included in this review were studies of rare cancers conducted by TCGA [39, 43–46, 48–56, 60, 93]. The studies included in this review which followed TCGA methodology all presented with high methodological rigour, usually with large sample numbers and even a broader range of participant ethnicity which is crucially needed in genomic analysis. With few methodological differences between research projects, these studies followed a comprehensive analytical pipeline which involved the generation and interpretation genomic, epigenomic, transcriptomic and proteomic data, providing a powerful impetus for standardised multi-omic methodology (Additional file 1, Table S1). This enabled researchers to identify molecular relationships between cancers, cluster prognostic variants and elucidate future therapeutic targets to explore. Furthermore, much of this anonymised data is publicly available on the Genomic Data Commons Data Portal for future research projects to utilise [146], and three non-TCGA cancer studies included in this review reported using this public data to overcome the rarity of their studied cancer type, to confirm cell ontology and even simply as a comparative control for their own gene expression data [69, 70, 99].

An additional point of interest was the computational algorithms used to overcome the statistical challenge of data integration in multi-omic studies of rare disease. Approaches to the analysis of multi-omic data vary dependent on research group preferences and bioinformatic experience, with many choosing to simply analyse each ‘omic’ dataset independently and identify overlapping molecular variation within top ranked genes (e.g. genes which show differentially methylated CpG sites from microarray analysis that correlate with differential gene expression from mRNA sequencing). However, this approach can lead to missing variants with biological significance which may not be immediately clear, for example missing a relationship between differentially methylated genes which could indirectly impact downstream protein production. Therefore, for those with bioinformatic expertise, integration of multi-omic data largely falls into three categories; early data integration, late data integration and statistical data integration, with a comprehensive description and examples of each provided by Rapport and Shamir, 2018 [147]. Early data integration involves the combining of integrated features from single omic data sets (concatenation) to output a single matrix representing similar features from multi-omic datasets in the participant, e.g. Autoencoder which has been used to integrate data from three omic analyses (DNA methylation, RNA-Seq and miRNA) for analysis of liver cancer [148]. Late data integration conducts clustering of related variants within single omic analysis and then integration of the single analyses clusters together, for example the Cluster-Of-Cluster-Assignments (CoCA) algorithm which looks across multiple omic analyses to define subclasses, whilst removing the need for data normalization prior to clustering and adding weight to analyses type so that large platforms do not dominant results (e.g. 450 K array compared to reverse phase protein array) [149]. Another example of late data integration tools is the similarity network fusion (SNF) which develops a network of patient level data rather than individual clusters enabling prognostic prediction [150]. Finally, statistical algorithms infer the most probable clusters within multi-omic datasets, for example PARADIGM which infers associations of molecular variants with patient phenotype by incorporating pathway activity and inactivity data [151]. Multi-Omics Factor Analysis (MOFA) is a second example of a statistical multi-omic data integration tool and an unsupervised model for identification of biological and technical variability [152].

In this scoping review we found that studies utilising multi-omic specific software to facilitate data integration across omics platforms comprised just 11% of included articles (7 studies). These included three algorithms developed by TCGA [1]; COCA consensus clustering (described above) [2] iCluster, an integrative multi-variate regression clustering algorithm which looks across several datatypes (DNA methylation, copy number variants (CNVs), mRNA and miRNA) to identify molecular patterns (also an example of a data integration technique which spans the criteria of both early and statistical integration), and [3] PARADIGM (described above) [39, 43, 45, 46, 49, 60]. In addition to studies which utilised TCGA specific algorithms, the only other study included in this review which discussed a bioinformatic pipe-line for multi-omic data integration was in vulvar carcinoma [81], in which the researchers used the CONEXIC algorithm to combine CNV and gene expression data to construct hypothesised regulatory networks, providing a ranked score which informs how well a particular variant predicts module behaviour, with high scores indicating high tumour adaptive advantage.

**Fig. 5:**
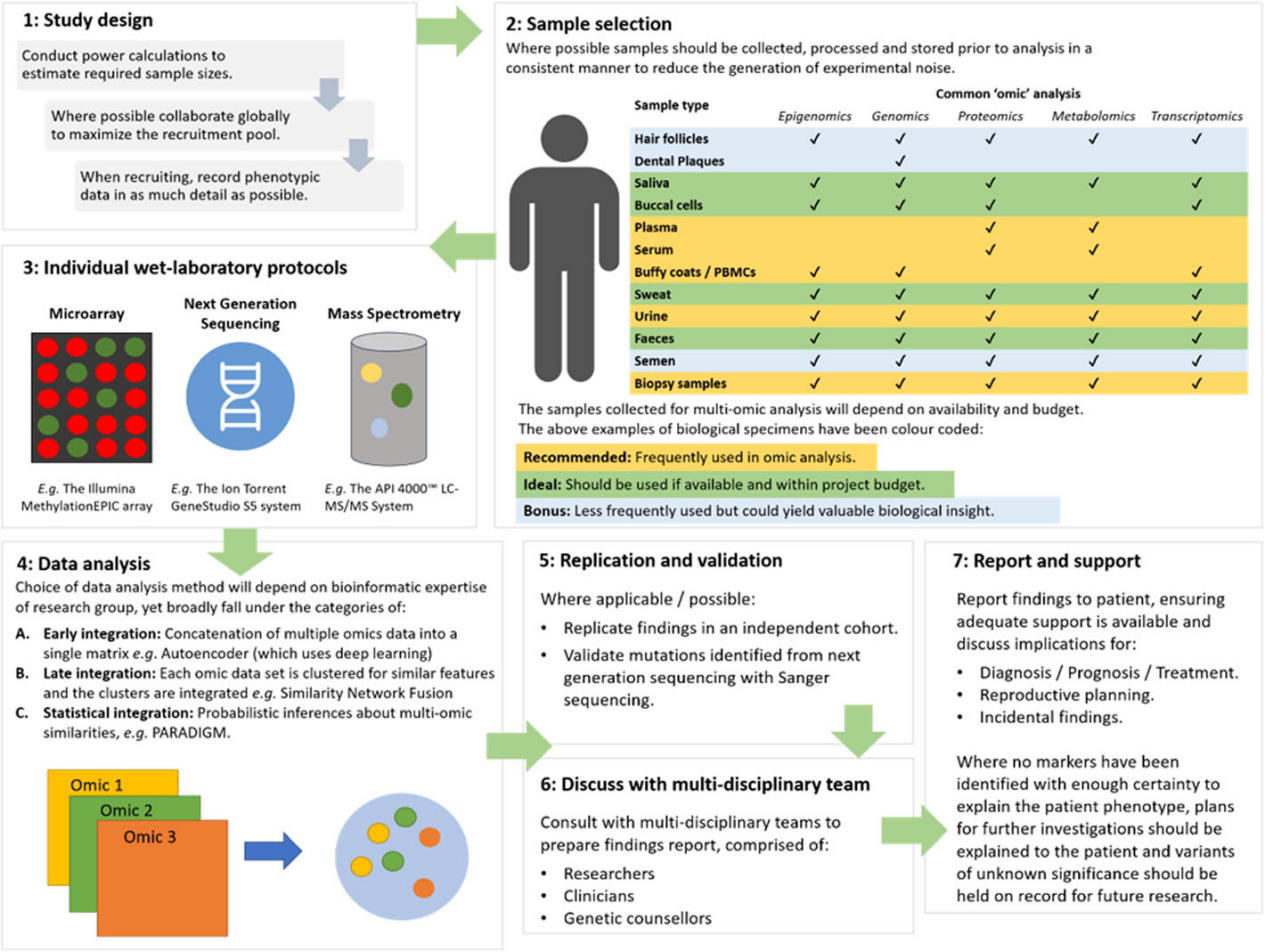
Proposed workflow for multi-omic analysis of rare diseases. To conduct an impactful study of multi-omics and rare disease, careful planning from study conceptualisation is crucial

The pipe-lines for laboratory and computational analysis described here focus primarily on cancer. Development of a similar integrative workflow for non-cancerous rare diseases, coupled with international collaboration to increase sample size, would be useful to increase pathogenic variant identification, diagnostic yield and development of a defined care pathway. Figure 5 illustrates a workflow which could be utilised for the planning and implementation of multi-omic rare disease studies when considering study design, selection of biological material for common omic analysis, data integration and reporting of findings to patients. Furthermore, there is a need for discussion on ensuring the data we generate is publicly available, whilst protecting patient confidentiality, to enable large scale collaborative efforts, a phenomenon which would be particularly helpful for diagnosing currently undiagnosed patients. Such a discussion and development of resources should involve continuous consultation with patients and their family members [16, 153].

### Multi-omics can provide a diagnosis to previously undiagnosed patients with rare phenotypes

Escaping the diagnostic odyssey is a major hurdle many patients with rare diseases face. This review highlighted studies which specifically intended to utilise multi-omics for provision of a diagnosis to previously undiagnosed rare phenotypes. Whilst most articles included in this paper sought to identify disease driving mutations, which could themselves be further investigated to elucidate a definitive molecular diagnosis, just three studies were identified with the specific aim of providing a diagnosis for patients with previously undiagnosed rare diseases through multi-omic analysis [58, 124, 136], as well as a further two conference abstracts for which no full text was available [134, 154].

Through a combination of comprehensive WES, chromosome microarray (CMA), linkage analysis and mRNA analysis, one study aimed to provide a diagnosis for a combination of the complex undiagnosed phenotypes: non-syndromic hearing loss (NSHL), aberrant skeletal phenotypes and significant developmental delay, in four individuals [124]. WES identified a recessively inherited splice variant in *PDZD7* (c.226 + 2_226 + 5delTAGG) likely to explain the NSHL phenotype, which was confirmed through mRNA analysis to inhibit gene expression in affected individuals, as no *PDZD7* exons were amplified. Furthermore, the developmental delay and microcephaly phenotype was explained via CMA through identification of a de novo unbalanced translocation in chromosome 8 and 18. The skeletal phenotype was associated with an autosomal dominantly inherited variant in *COL1A1* which lead to a diagnosis of osteogenesis imperfecta. This study reflects the phenotypic heterogeneity that is often present in undiagnosed rare diseases and demonstrates the utility of providing comprehensive genomic analysis with additional confirmatory mRNA analysis to maximise diagnostic yield. A second study aimed to utilise WGS, protein and mRNA analyses to aid the diagnosis of a family with heterogeneous myopathic and neurogenic phenotypes [136], uncovering five likely pathogenic exonic variants. Of these, a single mutation in the gene *NEFL* was identified in all affected family members, (c.1261C > T; p.R421X associated with truncated *NEFL* protein levels) which has previously been associated with Charcot-Marie-Tooth disease. This study is an excellent example of the power of multi-omic analysis to provide a molecular diagnosis for patients with rare undiagnosed phenotypes, while expanding on a previously known clinical phenotype, with both of the above studies utilising genomic analyses complemented by a form of transcriptomic analyses. Finally, a third small case-control study of eight patients, from four unrelated families, utilised WES and global metabolomics to identify diagnostic biomarkers of the rare disease mitochondrial aconitase deficiency [58]. The research team identified 758 metabolomic features with a minimum fold change of 1.5 between cases and controls, including α-ketoglutarate which was reduced 4.3 fold in ACO2 deficient patients and thus likely to contribute to the pathogenic phenotype. This study is the first to report a diagnostic biomarker of mitochondrial aconitase deficiency, using multi-omic technologies.

Further to the above studies, two conference abstracts were identified which also briefly discussed the utility of multi-omics for diagnosis of rare neuro-metabolic diseases [134, 154]. The first of these described utilising WES and metabolomic analysis of undiagnosed neuro-metabolic diseases in 59 individuals with a diagnostic yield of 43% [134]. However, unfortunately no full text is currently available for this article and little detail with regards to the target genes and metabolites identified is provided in the abstract. The second conference abstract also described the application of WES and metabolomics to aid diagnosis of complex rare phenotypes including neuro-metabolic diseases [154]. The researchers reported diagnosis of 179/500 previously undiagnosed individuals, with 8% of this diagnostic yield originating from metabolomic analysis alone, reflecting that a combined omics approach to diagnosis is indeed capable of greater diagnostic yield than WES alone.

### Pathogenic and prognostic markers can be identified by integration of multi-omic datasets

Elucidation of disease driving molecular profiles through integrative multi-omic analysis, most commonly genomic, epigenomic, transcriptomic, proteomic and metabolomic analysis, was the primary focus of most articles included in this review. Whilst it would be impractical to discuss each of the driver mutations identified for each rare disease studied within this review across all studied omic types, a comprehensive overview of pathogenic variants identified in individual studies is available for reference in Additional file 1: Table S1 (key results column). One example of note was a study of mevalonate kinase deficiency, a recessively inherited auto-inflammatory disorder, with multiple organ involvement. The spectrum of clinical presentations includes hyperimmunoglobulinemia D syndrome, periodic fever syndrome and mevalonic aciduria [155]. This study conducted WES, RNA-Seq and differential protein analysis in a case study of two sisters presenting with polarised phenotypic heterogeneity where they both harboured a known driver homozygous mutation in *MVK* but only one sibling presented with disease symptoms [111]. Their integrative multi-omic analysis identified a rare mutation in the modifier gene STAT1 resulting in upregulated mRNA AND protein expression, likely responsible for the phenotype in the affected sister. Single omic analysis alone was insufficient to detect this mutation, and therefore is exemplary of why these multi-omic studies are crucial in identifying a cause for rare diseases and explaining the phenotypic heterogeneity which can complicate patient care.

The identification of prognostic biomarkers through multi-omic analysis was highlighted in several studies of rare cancer including adrenocortical carcinoma (ACC), sarcoma, uveal melanoma and pseudomyxoma peritonei. Distinct prognostic groups of ACC were discussed in three of the five included ACC studies [39, 64, 65], including three prognostic molecular subtypes of ACC clustered by DNA methylation profile with 92.4% accuracy [39], two clusters by DNA methylation changes with frequent gene mutations (poor prognosis) and miRNA regulation (good prognosis) [64]. A third study reported the increased power of prognostic prediction accuracy using multi-omic data compared to singular analysis, specifically through the integration of several somatic variants, pathway analysis and differential methylation [65]. In a large case-control study of six different sarcoma subtypes, three prognostic clusters were identified through integration of somatic CNAs and DNA methylation data in dedifferentiated liposarcoma, in which the first two groups (*JUN* amplified and *TERT* amplified with chromosome instability) had a worse survival rate than the third cluster (6q25.1 amplified and less unbalanced chromosome segments), with *JUN* identified as a potential therapeutic target due to its overexpression previously being shown to increase tumour migration and invasion [49]. In a study of uveal melanoma, four molecularly distinct groups were identified with differences in prognostic outcomes: two associated with poor-prognosis, (monosomy 3) and two with better-prognosis, (disomy 3) [43]. Finally, one study of pseudomyxoma peritonei (a very rare form of appendix cancer) showed that aberrant p53 staining reflected a worse overall survival in patients compared to normal p53 staining (19% compared to 80% five year survival) [90].

### Multi-omic analysis can identify both novel treatments and drug re-purposing opportunities

Care for patients with rare diseases often relies on symptom management, rather than treatment of the underlying cause, with limited therapeutic options available. The most frequent age-group of participants in the studies included in this review was between 0 and 10 years, followed by a significant drop off until a peak again at age group of 41–50 years, which stresses the need for early diagnosis and therapeutic intervention to improve survival and quality of life for these children. Therefore, it is unsurprising that identification of promising novel therapeutic targets through multi-omic analysis was a consistently observed research aim across studies of non-cancerous rare diseases and rare cancers.

For example, one study identified 156 differentially methylated genes in ACC, including hypermethylation of *CYP1B1*, which was shown to have sensitivity to the methylation inhibitor decitabine in an ACC cell line. Furthermore, the same study found that cell proliferation occurred in the mutated genes *GATA6, G0S2, MEIS1, NCOA7, KCTD12, FAM1156A* following treatment with the oncology drug oncostatin M [41]. However even where novel therapeutic targets are identified as excellent candidates for clinical research, the expense of trials often results in pharmaceutical companies refusing to test and a produce a novel drug. For those novel drugs fortunate enough to be deemed worth the financial investment, the average timeframe from experimentation to clinical implementation is 12 years [156]. Therefore, re-purposing of drugs already approved for use in a different disease has become an increasing focus of the search for therapeutics in rare disease, in particular for precision oncology medicine [157]. This scoping review found that identification of drug repurposing opportunities to over-come the lack of treatment availability was a strong recurrent theme for the multi-omic analyses of rare cancer studies. For example the drug Ponatinib, which is used normally to treat leukaemia, was identified as a potential drug repurposing opportunity for small cell carcinoma of the ovary hypercalcaemic type, through integrated proteomic and transcriptomic analysis with functional cell-line and animal models [42]. Additionally, 16 potential novel ACC drug targets were identified for which there is varying degrees of evidence for drug targeting in other cancers targeting of the genes: *CDK4, NOTCH1, NF1, MDM2, EGFR, BRCA1, BRCA2, ATM, BRAF, PTCH1, TSC1, TSC2, KIT, RET, ESR1, EZH2* [65]. With this in mind, it would be useful to explore opportunities for drug repurposing via multi-omic analysis for non-cancerous rare diseases also.

## Conclusions

This scoping review highlights the exponential increase of multi-omic studies of rare diseases in the past decade, reflecting how the advent of NGS and high-density arrays have enabled multi-omic analysis. We have also highlighted in this review that the most frequently age group of participants identified was 0–10 years. This is concordant with the life expectancy of less than five years for a third of all rare disease patients, and emphases the importance of early diagnosis and implementation of a defined care pathway involving optimised treatment and not symptom management alone which can be provided by multi-omic analyses. Taken together, the discussed themes emphasise the need for the development of a standardised pipeline, to ensure unbiased and accurate reporting of biomarkers, as well as international collaboration to address the low participant numbers and biased participant ethnicity numbers which plague the power of rare disease research studies. The workflow provided in this review will be useful for researchers planning multi-omic studies of rare disease, whether that be for cancer or non-cancerous conditions. Projects such as the previously mentioned *100,000 Genomes Project*, and moving forward, the Five Million Genomes project, as well the NIH UDP and the IRDiRC, provide a platform for multi-omic analysis and are therefore fundamental for the future of rare disease research.

## Supplementary information

**Additional file 1: Table S1.** Full data extraction, sheet 2, abbreviations from Table S1.

**Additional file 2: Table S2.** Template quality appraisal form, **Table S3.** Articles excluded as not written in English but which may be relevant and utilised by researchers with translation resources available.

**Additional file 3: Table S4.** List of several multi-omics.

## Data Availability

This article is a review of publically available data.

## Abbreviations

ACC: Adrenocortical carcinoma
cDNA: Complementary DNA
CMA: Chromosome microarray
CNVs: Copy number variants
COCA: Cluster-Of-Cluster-Assignment
DNA: Deoxyribonucleic acid
HDRUK: Health Data Research UK
IRDiRC: International Rare Diseases Research Consortium
JBI: Joanna Briggs Institute
LC-MS/MS: Liquid chromatography with tandem mass spectrometry
miRNA: Microrna
MOFA: Multi-Omics Factor Analysis
mRNA: Messenger RNA
NGS: Next generation sequencing
NHS: National Health Service
NIH: National Institute of Health
NIHR: National Institute for Health Research
NSHL: Non-syndromic hearing loss
PCC: Population, concept, context
RNA: Ribonucleic acid
RNA-Seq: Ribonucleic acid sequencing
SNF: Similarity network fusion
TCGA: The Cancer Genome Atlas
UDP: Undiagnosed Diseases Program
UK: United Kingdom
US: United States
WES: Whole exome sequencing
WGS: Whole genome sequencing

## References

[1] RARE DISEASES - a major unmet medical need. Luxembourg: European Commission 2017.

[2] Global Genes. RARE Diseases: Facts and Statistics [Available from: https://globalgenes.org/rare-diseases-facts-statistics/. Accessed 9 Nov 2018.

[3] He J, Kang Q, Hu J, Song P, Jin C. China has officially released its first national list of rare diseases. Intractable Rare Dis Res. 7(2):145–7. 10.5582/irdr.2018.01056 PMC5982625 29862160

[4] Rare disease impact report: insights from patients and the medical community 2013. Shire; 2013.

[5] Esquivel-Sada D, Nguyen MT (2018). Diagnosis of rare diseases under focus: impacts for Canadian patients. J Community Genet.

[6] Crowe A, McAneney H, Morrison PJ, Cupples M, McKnight AJ. A quick reference guide for rare disease. Br J Gen Pract. 2020. In press. 10.3399/bjgp20X709853 PMC7194765 32354833

[7] McKnight AJ, on behalf of a collaborative team. Recommendation for a Collaborative Centre of Expertise for Rare Diseases in Northern Ireland (CERDNI). 2013.

[8] Brady AF, Demirdas S, Fournel-Gigleux S, Ghali N, Giunta C, Kapferer-Seebacher I (2017). The Ehlers-Danlos syndromes, rare types. Am J Med Genet C: Semin Med Genet.

[9] Girirajan S, Rosenfeld JA, Coe BP, Parikh S, Friedman N, Goldstein A (2012). Phenotypic heterogeneity of genomic disorders and rare copy-number variants. N Engl J Med.

[10] Martin-Sierra C, Gallego-Martinez A, Requena T, Frejo L, Batuecas-Caletrio A, Lopez-Escamez JA (2017). Variable expressivity and genetic heterogeneity involving DPT and SEMA3D genes in autosomal dominant familial Meniere's disease. Eur J Hum Genet.

[11] Kvarnung M, Nordgren A, MP DLP, Taruscio D, Groft SC (2017). Intellectual Disability & Rare Disorders: A Diagnostic Challenge. Rare Diseases Epidemiology: Update and Overview, 2nd Edition. Advances in Experimental Medicine and Biology.

[12] Giovannini M, Luzzati M, Ferrara G, Buccoliero AM, Simonini G, de Martino M (2018). Common symptoms for a rare disease in a girl with sarcoidosis: a case report. Ital J Pediatr.

[13] Posey JE, Harel T, Liu P, Rosenfeld JA, James RA, Coban Akdemir ZH (2017). Resolution of disease phenotypes resulting from multilocus genomic variation. N Engl J Med.

[14] HSCNI. Experience of Diagnosis, Views of patients and carers of diagnosis of rare disease in Northern Ireland. Patient Client Council. 2012. https://patientclientcouncil.hscni.net/download/19/reports/528/experience-of-diagnosis.pdf.

[15] de Vries E, Fransen L, van den Aker M, Meijboom BR (2018). Preventing gatekeeping delays in the diagnosis of rare diseases. Br J Gen Pract.

[16] Crowe AL, McKnight AJ, McAneney H (2019). Communication needs for individuals with rare diseases within and around the healthcare system of Northern Ireland. Front Public Health.

[17] Bradley J. ISCF HDRUK DIH Sprint Exemplar: Cloud-based integration of phenotype and genotype data for rare disease research 2019. Available from: https://europepmc.org/grantfinder/grantdetails?query=pi%3A%22Bradley%2BJ%22%2Bgid%3A%22MC_PC_18030%22%2Bga%3A%22Medical+Research+Council%22. Accessed 4 Dec 2019.

[18] Posey JE (2019). Genome sequencing and implications for rare disorders. Orphanet J Rare Dis.

[19] Turnbull C, Scott RH, Thomas E, Jones L, Murugaesu N, Pretty FB, et al. The 100 000 Genomes Project: Bringing whole genome sequencing to the NHS. BMJ (Online). 2018;361 (no pagination)(k1687). 10.1136/bmj.k1687 29691228

[20] Sivapalaratnam S, Bioresource N (2018). The rare diseases pilot for the 100,000 genomes project: findings in known and new genes by analysis of 3,549 whole genome sequenced samples from patients and relatives with Haematological. Haemostasis and Immune Disorders Blood.

[21] Matt Hancock announces ambition to map 5 million genomes: Department of Health and Social Care; 2018 [Available from: https://www.gov.uk/government/news/matt-hancock-announces-ambition-to-map-5-million-genomes. Accessed 4 Dec 2019.

[22] Chandrasekharan S, Minear MA, Hung A, Allyse M (2014). Noninvasive prenatal testing goes global. Sci Transl Med.

[23] International Rare Diseases Research Consortium IRDiRC; 2011. Available from: http://www.irdirc.org/about-us/vision-goals/. Accessed 12 Dec 2019.

[24] Tifft CJ, Adams DR (2014). The National Institutes of Health undiagnosed diseases program. Curr Opin Pediatr.

[25] Boycott KM, Hartley T, Biesecker LG, Gibbs RA, Innes AM, Riess O (2019). A diagnosis for all rare genetic diseases: the horizon and the next Frontiers. Cell..

[26] Alphabetically ordered list of omes and omics 2016. Available from: http://omics.org/index.php/Alphabetically_ordered_list_of_omes_and_omics. Accessed 15 Oct 2017.

[27] Frésard L, Smail C, Ferraro NM, Teran NA, Li X, Smith KS (2019). Identification of rare-disease genes using blood transcriptome sequencing and large control cohorts. Nat Med.

[28] Delavan B, Roberts R, Huang RL, Bao WJ, Tong WD, Liu ZC (2018). Computational drug repositioning for rare diseases in the era of precision medicine. Drug Discov Today.

[29] Lopez de Maturana E, Alonso L, Alarcon P, Martin-Antoniano IA, Pineda S, Piorno L, et al. Challenges in the Integration of Omics and Non-Omics Data. Genes (Basel). 2019;10(3):238. 10.3390/genes10030238 PMC6471713 30897838

[30] Kerr K, McAneney H, McKnight AJ (2019). Protocol for a scoping review of multi-omic analysis for rare diseases. BMJ Open.

[31] Tricco AC, Lillie E, Zarin W, O'Brien KK, Colquhoun H, Levac D (2018). PRISMA extension for scoping reviews (PRISMA-ScR): checklist and explanation. Ann Intern Med.

[32] EURODIS. About Rare Diseases. Available from: https://www.eurordis.org/about-rare-diseases. Accessed 15 Mar 2018.

[33] Gatta G, van der Zwan JM, Casali PG, Siesling S, Dei Tos AP, Kunkler I (2011). Rare cancers are not so rare: the rare cancer burden in Europe. Eur J Cancer.

[34] Greenlee RT, Goodman MT, Lynch CF, Platz CE, Havener LA, Howe HL (2010). The occurrence of rare cancers in U.S. adults, 1995–2004. Public Health Rep.

[35] TCGA. The Cancer Genome Atlas, Cancers Selected for Study: National Cancer Institute. Available from: https://www.cancer.gov/about-nci/organization/ccg/research/structural-genomics/tcga/studied-cancers. Accessed 13 Aug 2019.

[36] Popay J, Roberts H, Sowden A, Petticrew M, Arai L, Rodgers M (2006). Guidance on the conduct of narrative synthesis in systematic reviews: a product from the ESRC methods Programme.

[37] Armstrong R, Hall BJ, Doyle J, Waters E (2011). ‘Scoping the scope’ of a cochrane review. J Public Health.

[38] Papuc SM, Abela L, Steindl K, Begemann A, Simmons TL, Schmitt B (2019). The role of recessive inheritance in early-onset epileptic encephalopathies: a combined whole-exome sequencing and copy number study. Eur J Hum Genet.

[39] Zheng S, Cherniack AD, Dewal N, Moffitt RA, Danilova L, Murray BA (2016). Comprehensive pan-genomic characterization of adrenocortical Carcinoma. Cancer Cell.

[40] Di Michele M, Goubau C, Waelkens E, Thys C, Overbergh L, Buyse G (2013). Functional studies and proteomics in platelets and fibroblasts reveal a lysosomal defect with increased cathepsin-dependent apoptosis in ATP1A3 defective alternating hemiplegia of childhood. J Thromb Haemost.

[41] Gara SK, Wang Y, Patel D, Liu-Chittenden Y, Jain M, Boufraqech M (2015). Integrated genome-wide analysis of genomic changes and gene regulation in human adrenocortical tissue samples. Nucleic Acids Res.

[42] Lang JD, Hendricks WPD, Orlando KA, Yin H, Kiefer J, Ramos P (2018). Ponatinib shows potent antitumor activity in small cell Carcinoma of the ovary Hypercalcemic type (SCCOHT) through multikinase inhibition. Clin Cancer Res.

[43] Hoadley KA, Yau C, Hinoue T, Wolf DM, Lazar AJ, Drill E (2018). Cell-of-Origin Patterns Dominate the Molecular Classification of 10,000 Tumors from 33 Types of Cancer. Cell.

[44] Ley TJ, Miller C, Ding L, Raphael BJ, Mungall AJ, Robertson A (2013). Genomic and epigenomic landscapes of adult de novo acute myeloid leukemia. N Engl J Med.

[45] Farshidfar F, Zheng S, Gingras M-C, Newton Y, Shih J, Robertson AG (2017). Integrative genomic analysis of Cholangiocarcinoma identifies distinct IDH-mutant molecular profiles. Cell Rep.

[46] Hmeljak J, Sanchez-Vega F, Hoadley KA, Shih J, Stewart C, Heiman D (2018). Integrative molecular characterization of malignant pleural mesothelioma. Cancer Discovery.

[47] Bell D, Berchuck A, Birrer M, Chien J, Cramer DW, The Cancer Genome Atlas Research N (2011). Integrated genomic analyses of ovarian carcinoma. Nature.

[48] Fishbein L, Leshchiner I, Walter V, Danilova L, Robertson AG, Johnson AR (2017). Comprehensive molecular characterization of Pheochromocytoma and Paraganglioma. Cancer Cell.

[49] Abeshouse A, Adebamowo C, Adebamowo SN, Akbani R, Akeredolu T, Ally A (2017). Comprehensive and Integrated Genomic Characterization of Adult Soft Tissue Sarcomas. Cell.

[50] Shen H, Shih J, Hollern DP, Wang L, Bowlby R, Tickoo SK (2018). Integrated molecular characterization of testicular germ cell tumors. Cell Rep.

[51] Radovich M, Pickering CR, Felau I, Ha G, Zhang HL, Jo H (2018). The Integrated Genomic Landscape of Thymic Epithelial Tumors. Cancer Cell.

[52] Cherniack AD, Shen H, Walter V, Stewart C, Murray BA, Bowlby R (2017). Integrated molecular characterization of uterine Carcinosarcoma. Cancer Cell.

[53] Levine DA, Getz G, Gabriel SB, Cibulskis K, Lander E, The Cancer genome atlas research N (2013). Integrated genomic characterization of endometrial carcinoma. Nature.

[54] Robertson AG, Shih J, Yau C, Gibb EA, Oba J, Mungall KL (2017). Integrative Analysis Identifies Four Molecular and Clinical Subsets in Uveal Melanoma. Cancer Cell.

[55] Davis Caleb F, Ricketts CJ, Wang M, Yang L, Cherniack Andrew D, Shen H (2014). The somatic genomic landscape of Chromophobe renal cell Carcinoma. Cancer Cell.

[56] Agrawal N, Akbani R, Aksoy BA, Ally A, Arachchi H, Asa Sylvia L (2014). Integrated genomic characterization of papillary thyroid Carcinoma. Cell..

[57] Crowther LM, Poms M, Plecko B (2018). Multiomics tools for the diagnosis and treatment of rare neurological disease. J Inherit Metab Dis.

[58] Abela L, Spiegel R, Crowther LM, Klein A, Steindl K, Papuc SM (2017). Plasma metabolomics reveals a diagnostic metabolic fingerprint for mitochondrial aconitase (ACO2) deficiency. PloS one.

[59] Steele CD, Tarabichi M, Oukrif D, Webster AP, Ye H, Fittall M, et al. Undifferentiated Sarcomas Develop through Distinct Evolutionary Pathways. Cancer Cell. 2019;35(3):441–56. 10.1016/j.ccell.2019.02.002 PMC6428691 30889380

[60] Hoadley KA, Yau C, Wolf DM, Cherniack AD, Tamborero D, Ng S (2014). Multiplatform analysis of 12 cancer types reveals molecular classification within and across tissues of origin. Cell..

[61] Munn Z, Peters MDJ, Stern C, Tufanaru C, McArthur A, Aromataris E (2018). Systematic review or scoping review? Guidance for authors when choosing between a systematic or scoping review approach. BMC Med Res Methodol.

[62] Fey MF, Buske C (2013). Acute myeloblastic leukaemias in adult patients: ESMO Clinical Practice Guidelines for diagnosis, treatment and follow-up. Ann Oncol.

[63] Fassnacht M, Dekkers OM, Else T, Baudin E, Berruti A, RRd K, et al. European Society of Endocrinology Clinical Practice Guidelines on the management of adrenocortical carcinoma in adults, in collaboration with the European Network for the Study of Adrenal Tumors. 2018;179(4):G1. 10.1530/EJE-18-0608 30299884

[64] Assie G, Letouze E, Fassnacht M, Jouinot A, Luscap W, Barreau O (2014). Integrated genomic characterization of adrenocortical carcinoma. Nat Genet.

[65] Lippert J, Appenzeller S, Liang R, Sbiera S, Kircher S, Altieri B (2018). Targeted molecular analysis in adrenocortical carcinomas: a strategy toward improved personalized prognostication. J Clin Endocrinol Metab.

[66] Thyparambil SP, Kim YJ, Chambers A, Yan D, Sellappan S, Gong C, et al. Comprehensive proteomic and genomic profiling to identify therapeutic targets in adenoid cystic carcinoma. J Clin Oncol Conference. 2018;36(15 Supplement 1).

[67] Global, regional, and national burden of brain and other CNS cancer, 1990–2016: a systematic analysis for the Global Burden of Disease Study 2016. Lancet Neurol. 2019;18(4):376–93. 10.1016/S1474-4422(18)30468-X PMC6416167 30797715

[68] Barkhoudarian G, Wang XA, Salomon M, Marzese D, Hua WH, Kelly DF, et al. Genetic and epigenetic alterations between pituitary adenoma and pituitary carcinoma. J Neurol Surg B Skull. 2017;78(Supplement 1).

[69] Classe M, Yao H, Mouawad R, Creighton CJ, Burgess A, Allanic F (2018). Integrated Multi-omic Analysis of Esthesioneuroblastomas Identifies Two Subgroups Linked to Cell Ontogeny. Cell Reports.

[70] Koh Y, Park I, Sun CH, Lee S, Yun H, Park CK (2015). Detection of a distinctive genomic signature in Rhabdoid Glioblastoma, a rare disease entity identified by whole exome sequencing and whole Transcriptome sequencing. Transl Oncol.

[71] Wang LH, Yamaguchi S, Burstein MD, Terashima K, Chang K, Ng HK (2014). Novel somatic and germline mutations in intracranial germ cell tumours. Nature.

[72] Kirstein MM, Vogel A (2016). Epidemiology and risk factors of Cholangiocarcinoma. Visc Med.

[73] Tilly H, Aurer I, Johnson P, Lenz G, Minard V, Ribrag V (2016). Diffuse large B-cell lymphoma and Burkitt lymphoma in adults and children. In: the European Hematology Association roadmap for European hematology research: a consensus document. Haematologica..

[74] Fibrolamellar Carcinoma: National Organization for Rare Disorders (NORD); 2019. Available from: https://rarediseases.org/rare-diseases/fibrolamellar-carcinoma/. Accessed 13 Aug 2019.

[75] Sorenson EC, Khanin R, Bamboat ZM, Cavnar MJ, Kim TS, Sadot E, et al. Genome and transcriptome profiling of fibrolamellar hepatocellular carcinoma demonstrates p53 and IGF2BP1 dysregulation. Plos One. 2017;12(5):e0176562. 10.1371/journal.pone.0176562 PMC5423588 28486549

[76] Makuuchi R, Terashima M, Kusuhara M, Nakajima T, Serizawa M, Hatakeyama K (2017). Comprehensive analysis of gene mutation and expression profiles in neuroendocrine carcinomas of the stomach. Biomed Res.

[77] Boikos SA, Pappo AS, Killian JK, LaQuaglia MP, Weldon CB, George S (2016). Molecular subtypes of KIT/PDGFRA wild-type gastrointestinal stromal tumors a report from the National Institutes of Health gastrointestinal stromal tumor clinic. Jama Oncology.

[78] Brooks SE, Zhan M, Cote T, Baquet CR (2004). Surveillance, epidemiology, and end results analysis of 2677 cases of uterine sarcoma 1989-1999. Gynecol Oncol.

[79] Casey MJ, Crotzer D. Cancer, Endometrial StatPearls; 2019. Available from: https://www.ncbi.nlm.nih.gov/books/NBK525981/. Accessed 15 Aug 2019.

[80] Cancer Stat Facts: Vulvar Cancer: National Cancer Institute. Available from: https://seer.cancer.gov/statfacts/html/vulva.html. Accessed 15 Aug 2019.

[81] Lavorato-Rocha AM, Akagi EM, Maia BD, Rodrigues IS, Botelho MCS, Marchi FA (2016). An integrative approach uncovers biomarkers that associate with clinically relevant disease outcomes in vulvar Carcinoma. Mol Cancer Res.

[82] Nuyts V, Nawrot T, Nemery B, Nackaerts K (2018). Hotspots of malignant pleural mesothelioma in Western Europe. Translational Lung Cancer Research.

[83] Cancer Stat Facts: Esophageal Cancer: National Cancer Institute. Available from: https://seer.cancer.gov/statfacts/html/esoph.html. Accessed 16 Aug 2019.

[84] Berends AMA, Buitenwerf E, de Krijger RR, Veeger NJGM, van der Horst-Schrivers ANA, Links TP (2018). Incidence of pheochromocytoma and sympathetic paraganglioma in the Netherlands: a nationwide study and systematic review. Eur J Intern Med.

[85] Mishra SP, Tiwary SK, Mishra M, Khanna AK (2013). Phyllodes tumor of breast: a review article. ISRN Surg.

[86] Jardim DLF, Conley A, Subbiah V. Comprehensive characterization of malignant phyllodes tumor by whole genomic and proteomic analysis: biological implications for targeted therapy opportunities. Orphanet J Rare Dis. 2013;8:112. 10.1186/1750-1172-8-112 PMC3751902 23895135

[87] Rare and less common cancers. In: Network NCI, editor. Incidence and Mortality in England, 2010 to 2013. England: Public Health England; 2015.

[88] Mehra R, Vats P, Kalyana-Sundaram S, Udager AM, Roh M, Alva A (2014). Primary urethral clear-cell adenocarcinoma: comprehensive analysis by surgical pathology, cytopathology, and next-generation sequencing. Am J Pathol.

[89] Smeenk R, Van Velthuysen M-L, Verwaal V, Zoetmulder FAN (2008). Appendiceal neoplasms and Pseudomyxoma Peritonei: a population based study. Eur J Surg Oncol.

[90] Nummela P, Saarinen L, Thiel A, Jarvinen P, Lehtonen R, Lepisto A (2015). Genomic profile of pseudomyxoma peritonei analyzed using next-generation sequencing and immunohistochemistry. Int J Cancer.

[91] Chedgy ECP, Vandekerkhove G, Herberts C, Annala M, Donoghue AJ, Sigouros M (2018). Biallelic tumour suppressor loss and DNA repair defects in de novo small-cell prostate carcinoma. J Pathol.

[92] Puca L, Bareja R, Prandi D, Shaw R, Benelli M, Karthaus WR, et al. Patient derived organoids to model rare prostate cancer phenotypes. Nat Commun. 2018;9:2404. 10.1038/s41467-018-04495-z PMC6008438 29921838

[93] Ko JJ, Grewal JK, Ng T, Lavoie JM, Thibodeau ML, Shen Y, et al. Whole-genome and transcriptome profiling of a metastatic thyroid-like follicular renal cell carcinoma. Cold Spring Harb Mol Case Stud. 2018;4(6) (no pagination)(a003137). 10.1101/mcs.a003137 PMC6318773 30446580

[94] Izabela Kordzińska-Cisek LG-S (2018). Salivary gland cancer — epidemiology. J Oncol.

[95] Qiu WL, Tong GX, Turk AT, Close LG, Caruana S, Su GH. Oncogenic PIK3CA mutation and Dysregulation in human salivary duct Carcinoma. Biomed Res Int. 2014;2014:810487. 10.1155/2014/810487 PMC3910486 24511546

[96] Burningham Z, Hashibe M, Spector L, Schiffman JD (2012). The epidemiology of sarcoma. Clinical sarcoma research.

[97] Hong AL, Tseng YY, Cowley GS, Jonas O, Cheah JH, Kynnap BD (2016). Integrated genetic and pharmacologic interrogation of rare cancers. Nat Commun.

[98] Kim JH, Megquier K, Sarver AL, Thomas R, Wang C, Elvers I, et al. Mutational and transcriptomic profiling identify distinct angiogenic and inflammatory subtypes of angiosarcoma. Cancer Res Conference. 2018;78(13 Supplement 1).

[99] Orth MF, Gerke JS, Knosel T, Altendorf-Hofmann A, Musa J, Alba-Rubio R (2019). Functional genomics identifies AMPD2 as a new prognostic marker for undifferentiated pleomorphic sarcoma. Int J Cancer.

[100] Samimi S, Rook AH, Kim EJ (2013). Update on Epidemiology of Cutaneous T-Cell Lymphoma. Epidemiology (Cambridge, Mass).

[101] Sekulic A, Liang WS, Tembe W, Izatt T, Kruglyak S, Kiefer JA (2015). Personalized treatment of Sezary syndrome by targeting a novel CTLA4: CD28 fusion. Molecular genetics & genomic medicine.

[102] Shanmugalingam T, Soultati A, Chowdhury S, Rudman S, Van Hemelrijck M (2013). Global incidence and outcome of testicular cancer. Clin Epidemiol.

[103] Girard N, Ruffini E, Marx A, on behalf of the EGC, on behalf of the EGC, on behalf of the EGC (2015). Thymic epithelial tumours: ESMO Clinical Practice Guidelines for diagnosis, treatment and follow-up†. Ann Oncol.

[104] Saito K, Kobayashi E, Yoshida A, Araki Y, Kubota D, Tanzawa Y (2017). Angiomatoid fibrous histiocytoma: a series of seven cases including genetically confirmed aggressive cases and a literature review. BMC Musculoskelet Disord.

[105] La Vecchia C, Malvezzi M, Bosetti C, Garavello W, Bertuccio P, Levi F (2015). Thyroid cancer mortality and incidence: a global overview. Int J Cancer.

[106] Mancikova V, Montero-Conde C, Perales-Paton J, Fernandez A, Santacana M, Jodkowska K (2017). Multilayer OMIC data in medullary thyroid Carcinoma identifies the STAT3 pathway as a potential therapeutic target in RETM918T tumors. Clin Cancer Res.

[107] Mahendraraj K, Lau CS, Lee I, Chamberlain RS (2016). Trends in incidence, survival, and management of uveal melanoma: a population-based study of 7,516 patients from the surveillance, epidemiology, and end results database (1973-2012). Clin Ophthalmol.

[108] Barnes DJ, Hookway E, Athanasou N, Takeshi K, Oppermann U, Hughes S (2016). A germline mutation of CDKN2A and a novel RPLP1-C19MC fusion detected in a rare melanotic neuroectodermal tumor of infancy: a case report. BMC Cancer.

[109] Cohen S, Hyer W, Mas E, Auth M, Attard TM, Spalinger J (2019). Management of Juvenile Polyposis Syndrome in children and adolescents: a position paper from the ESPGHAN polyposis working group. J Pediatr Gastroenterol Nutr.

[110] Woodford-Richens KL, Rowan AJ, Poulsom R, Bevan S, Salovaara R, Aaltonen LA (2001). Comprehensive analysis of SMAD4 mutations and protein expression in juvenile polyposis - evidence for a distinct genetic pathway and polyp morphology in SMAD4 mutation carriers. Am J Pathol.

[111] Carapito R, Carapito C, Morlon A, Paul N, Vaca Jacome AS, Alsaleh G, et al. Multi-OMICS analyses unveil STAT1 as a potential modifier gene in mevalonate kinase deficiency. Ann Rheum Dis. 2018;77(11):1675–87. 10.1136/annrheumdis-2018-213524 PMC6225799 30030262

[112] Hara Y, Kawasaki N, Hirano KI, Hashimoto Y, Adachi J, Watanabe S, et al. Quantitative proteomic analysis of cultured skin fibroblast cells derived from patients with triglyceride deposit cardiomyovasculopathy. Orphanet J Rare Dis. 2013;8(1) (no pagination)(197). 10.1186/1750-1172-8-197 PMC3891998 24360150

[113] Turleau C (2008). Monosomy 18p. Orphanet J Rare Dis.

[114] Balog J, Goossens R, Lemmers RJLF, Straasheijm KR, Van Der Vliet PJ, Heuvel AVD, et al. Monosomy 18p is a risk factor for facioscapulohumeral dystrophy. J Med Genet. 2018;11:469–78. 10.1136/jmedgenet-2017-105153 PMC6019561 29563141

[115] M Ehrlich KJ, CMR Weemaes. ICF syndrome, ORPHA:2268 2006. Available from: https://www.orpha.net/consor/cgi-bin/OC_Exp.php?Expert=2268&lng=EN. Accessed 14 Nov 2019.

[116] Grimbacher B, ERW P (2014). The European Society for Immunodeficiencies (ESID) registry 2014. Clin Exp Immunol.

[117] Gatto S, Gagliardi M, Franzese M, Leppert S, Papa M, Cammisa M (2017). ICF-specific DNMT3B dysfunction interferes with intragenic regulation of mRNA transcription and alternative splicing. Nucleic Acids Res.

[118] Hambleton S, Goodbourn S, Young DF, Dickinson P, Mohamad SMB, Valappil M (2013). STAT2 deficiency and susceptibility to viral illness in humans. Proc Natl Acad Sci U S A.

[119] Zennaro D, Scala E, Pomponi D, Caprini E, Arcelli D, Gambineri E (2012). Proteomics plus genomics approaches in primary immunodeficiency: the case of immune dysregulation, polyendocrinopathy, enteropathy, X-linked (IPEX) syndrome. Clin Exp Immunol.

[120] Chang IJ, He M, Lam CT (2018). Congenital disorders of glycosylation. Ann Transl Med.

[121] Stray-Pedersen A, Backe PH, Sorte HS, Morkrid L, Chokshi NY, Erichsen HC (2014). PGM3 mutations cause a congenital disorder of glycosylation with severe immunodeficiency and skeletal dysplasia. Am J Hum Genet.

[122] Kohlhase J. Townes-Brocks syndrome, ORPHA:857: Orpha.net; 2013. Available from: https://www.orpha.net/consor/cgi-bin/Disease_Search.php?lng=EN&data_id=218&Disease_Disease_Search_diseaseGroup=Townes-brock-syndrome&Disease_Disease_Search_diseaseType=Pat&Disease(s)/group%20of%20diseases=Townes-Brocks-syndrome&title=Townes-Brocks%20syndrome&search=Disease_Search_Simple. Accessed 14 Nov 2019.

[123] Primrose syndrome: GARD Genetic and Rare Diseases Information Center; 2016. Available from: https://rarediseases.info.nih.gov/diseases/4488/primrose-syndrome. Accessed 14 Nov 2019.

[124] Le Quesne SP, James C, Ocaka L, Tekman M, Grunewald S, Clement E, et al. An example of the utility of genomic analysis for fast and accurate clinical diagnosis of complex rare phenotypes. Orphanet J Rare Dis. 2017;12(1):24. 10.1186/s13023-017-0582-8 PMC5297239 28173822

[125] Bozal-Basterra L, Martin-Ruiz I, Pirone L, Liang Y, Sigursson JO, Gonzalez-Santamarta M, et al. Truncated SALL1 Impedes Primary Cilia Function in Townes-Brocks Syndrome. Am J Hum Genet. 2018;102(2):249–65. 10.1016/j.ajhg.2017.12.017 PMC5985538 29395072

[126] Liu YC, Li Y, March ME, Kenny N, Xu KX, Wang FX, et al. Copy number variation in CEP57L1 predisposes to congenital absence of bilateral ACL and PCL ligaments. Human Genomics. 2015;9:31. 10.1186/s40246-015-0053-z PMC4642759 26561035

[127] De Lonlay P. Hereditary myopathy with lactic acidosis due to ISCU deficiency, ORPHA:43115: orpha.net; 2008. Available from: https://www.orpha.net/consor/cgi-bin/Disease_Search.php?lng=EN&data_id=10579&Disease_Disease_Search_diseaseGroup=Aconitase-deficiency&Disease_Disease_Search_diseaseType=Pat&Disease(s)/group%20of%20diseases=Hereditary-myopathy-with-lactic-acidosis-due-to-ISCU-deficiency&title=Hereditary%20myopathy%20with%20lactic%20acidosis%20due%20to%20ISCU%20deficiency&search=Disease_Search_Simple. Accessed 14 Nov 2019.

[128] Leoncini E, Baranello G, Orioli IM, Anneren G, Bakker M, Bianchi F (2008). Frequency of holoprosencephaly in the international clearinghouse birth defects surveillance systems: searching for population variations. Birth Defects Res A Clin Mol Teratol.

[129] Wexler NS, Collett L, Wexler AR, Rawlins MD, Tabrizi SJ, Douglas I (2016). Incidence of adult Huntington's disease in the UK: a UK-based primary care study and a systematic review. BMJ Open.

[130] Abela L, Simmons L, Steindl K, Schmitt B, Mastrangelo M, Joset P (2016). N 8-acetylspermidine as a potential plasma biomarker for Snyder-Robinson syndrome identified by clinical metabolomics. J Inherit Metab Dis.

[131] Jany PL, Hagemann TL, Messing A (2013). GFAP expression as an indicator of disease severity in mouse models of Alexander disease. Asn Neuro.

[132] Kim A, Savary C, Dubourg C, Carre W, Mouden C, Hamdi-Roze H (2018). Integrated clinical and omics approach to rare diseases: novel genes and oligogenic inheritance in holoprosencephaly. Brain.

[133] Siebzehnrubl FA, Raber KA, Urbach YK, Schulze-Krebs A, Canneva F, Moceri S (2018). Early postnatal behavioral, cellular, and molecular changes in models of Huntington disease are reversible by HDAC inhibition. Proc Natl Acad Sci U S A.

[134] Van Karnebeek CD, Salvarinova R, Horvath G, Stockler S, Vallance H, Sinclair G (2016). Diagnosis and discovery of treatable neurometabolic diseases via an integrated-omics approach. J Inherited Metabolic Dis.

[135] Abstracts. Molecular Genetics and Metabolism. 2018;123(3):185–284.

[136] Agrawal PB, Joshi M, Marinakis NS, Schmitz-Abe K, Ciarlini P, Sargent JC (2014). Expanding the phenotype associated with the NEFL mutation neuromuscular disease in a family with overlapping Myopathic and neurogenic findings. Jama Neurology.

[137] Tambo FFM, Tolefac PN, Ngowe MN, Minkande JZ, Mbouche L, Guemkam G (2018). Posterior urethral valves: 10 years audit of epidemiologic, diagnostic and therapeutic aspects in Yaoundé gynaeco-obstetric and paediatric hospital. BMC Urol.

[138] Buffin-Meyer B, Klein J, Breuil B, Muller F, Moulos P, Groussolles M (2018). Combination of the fetal urinary metabolome and peptidome for the prediction of postnatal renal outcome in fetuses with PUV. J Proteome.

[139] Leinoe E, Zetterberg E, Kinalis S, Ostrup O, Kampmann P, Norstrom E (2017). Application of whole-exome sequencing to direct the specific functional testing and diagnosis of rare inherited bleeding disorders in patients from the Oresund region, Scandinavia. Br J Haematol.

[140] Cummings BB, Marshall JL, Tukiainen T, Lek M, Donkervoort S, Foley AR, et al. Improving genetic diagnosis in Mendelian disease with transcriptome sequencing. Sci Transl Med. 2017;9(386). 10.1126/scitranslmed.aal5209 PMC5548421 28424332

[141] Merker JD, Wenger AM, Sneddon T, Grove M, Zappala Z, Fresard L (2018). Long-read genome sequencing identifies causal structural variation in a Mendelian disease. Genetics Med.

[142] Mantere T, Kersten S, Hoischen A. Long-Read Sequencing Emerging in Medical Genetics. Front Genet. 2019;10(426). https://www.ncbi.nlm.nih.gov/pubmed/31134132. 10.3389/fgene.2019.00426 PMC6514244 31134132

[143] Mitsuhashi S, Matsumoto N. Long-read sequencing for rare human genetic diseases. J Hum Genet. 2019;65(1):11–9. 10.1038/s10038-019-0671-8 31558760

[144] Bayega A, Wang YC, Oikonomopoulos S, Djambazian H, Fahiminiya S, Ragoussis J (1783). Transcript profiling using long-read sequencing technologies. Methods Mol Biol.

[145] Gigante S, Gouil Q, Lucattini A, Keniry A, Beck T, Tinning M (2019). Using long-read sequencing to detect imprinted DNA methylation. Nucleic Acids Res.

[146] Genomic Data Commons Data Portal: National Cancer Institute 2019. Available from: https://portal.gdc.cancer.gov. Accessed 14 Nov 2019.

[147] Rappoport N, Shamir R (2018). Multi-omic and multi-view clustering algorithms: review and cancer benchmark. Nucleic Acids Res.

[148] Chaudhary K, Poirion OB, Lu L, Garmire LX (2018). Deep learning-based multi-Omics integration robustly predicts survival in liver Cancer. Clinical cancer research : an official journal of the American Association for Cancer Research.

[149] The Cancer Genome Atlas Network. Comprehensive molecular portraits of human breast tumours. Nature. 2012;490(7418):61–70. 10.1038/nature11412 PMC3465532 23000897

[150] Wang B, Mezlini AM, Demir F, Fiume M, Tu Z, Brudno M (2014). Similarity network fusion for aggregating data types on a genomic scale. Nat Methods.

[151] Vaske CJ, Benz SC, Sanborn JZ, Earl D, Szeto C, Zhu J (2010). Inference of patient-specific pathway activities from multi-dimensional cancer genomics data using PARADIGM. Bioinformatics..

[152] Argelaguet R, Velten B, Arnol D, Dietrich S, Zenz T, Marioni JC (2018). Multi-Omics factor analysis-a framework for unsupervised integration of multi-omics data sets. Mol Syst Biol.

[153] Courbier S, Dimond R, Bros-Facer V (2019). Share and protect our health data: an evidence based approach to rare disease patients’ perspectives on data sharing and data protection - quantitative survey and recommendations. Orphanet J Rare Dis.

[154] Liu N, Raina R, Hubert L, Alaimo JP, Yang Y, Xiao J (2018). Combining broad-scale untargeted metabolomic profiling and whole exome sequencing technologies improves diagnosis of inherited metabolic disorders. Mol Genet Metab.

[155] Favier LA, Schulert GS (2016). Mevalonate kinase deficiency: current perspectives. Appl Clin Genet.

[156] Mohs RC, Greig NH (2017). Drug discovery and development: role of basic biological research. Alzheimers Dement (N Y).

[157] Pantziarka P, Meheus L (2018). Omics-driven drug repurposing as a source of innovative therapies in rare cancers. Exp Opinion Orphan Drugs.

