# Supplementary material for: A scoping review and proposed workflow for multi-omic rare disease research
Source: Orphanet J Rare Dis. 2020 Apr 28;15:107. doi: 10.1186/s13023-020-01376-x (PMC7189570; doi:10.1186/s13023-020-01376-x)
Supplement: Supplementary file 2 — Additional file 2: Table S2. Template quality appraisal form, Table S3. Articles excluded as not written in English but which may be relevant and utilised by researchers with translation resources available. [file 13023_2020_1376_MOESM2_ESM.docx]

**Table S2.** Template quality appraisal form.

|  |  | Yes | No | Unclear | Not Applicable | Comments |
| --- | --- | --- | --- | --- | --- | --- |
| 1 | Was the primary focus of the paper methylation? |  |  |  |  |  |
| 2 | Were the groups comparable other than the presence of disease in cases or the absence of disease in controls? |  |  |  |  |  |
| 3 | Were clinical notes such as patient history/current condition, age, sex and ethnicity reported? |  |  |  |  |  |
| 4 | Were cases and controls matched appropriately? |  |  |  |  |  |
| 5 | Was methylation measured in in a standard reliable way? |  |  |  |  |  |
| 6 | Was methylation measured in the same way for cases and controls? |  |  |  |  |  |
| 7 | Were confounding factors identified? |  |  |  |  |  |
| 8 | Were strategies to deal with confounding factors identified? |  |  |  |  |  |
| 9 | Was the exposure period of interest long enough to be meaningful? |  |  |  |  |  |
| 10 | What statistical analysis was used and was this analysis appropriate? |  |  |  |  |  |
| 11 | Were experimental controls used? |  |  |  |  |  |

**Table S3.** Articles excluded as not written in English but which may be relevant and utilised by researchers with translation resources available.

| 1. Takahashi K. The cutting edge of sarcoma genomics. [Japanese]. *Japanese Journal of Cancer and Chemotherapy* 2018;45(4):605-11. |
| --- |
| 2. Takahashi K. [The Cutting Edge of Sarcoma Genomics]. *Gan to Kagaku Ryoho [Japanese Journal of Cancer & Chemotherapy]*;45(4):605-11. |
| 3. Verloes A, Heron D, Billette de Villemeur T, et al. Diagnostic investigations for an unexplained developmental disability. [French]. *Archives de Pediatrie* 2012;19(2):194-207. |
| 4. Czlrjak L, Kiss CG, Kiss E. Does the number of patients with autoimmune disorders and the frequency of autoimmune diseases increase?. [Hungarian]. *Orvosi Hetilap* 2007;148(SUPPL. 1):17-20. |
| 5. Pelegrino Souza P, Sampaio M, Geber S. Epigenetic imbalance within genomic expression in human desease and ART. [Portuguese]. *Jornal Brasileiro de Reproducao Assistida* 2006;10(2):25-28. |
| 6. Dufke A, Riess O. Genomic imprinting - Is there a risk after IVF and ICSI?. [German]. *Journal fur Reproduktionsmedizin und Endokrinologie* 2004;1(1):28-32. |
| 7. Simon-Bouy B, Caron O. [Genomic medicine for every patient: Dream or reality?]. *Gynecologie, obstetrique, fertilite & senologie*;45(4):187-89. |
| 8. Mandel JL. Genomic revolution of rare disease diagnosis. [French]. *Presse Med* 2012;41(SUPPL.1):S26-S28. |
| 9. JIA J, MING Y, AN Z, et al. A multi-omics annotation platform for rare disease to benefit precision medicine. *SCIENTIA SINICA Vitae* 2018;48(9):1026-32. |
| 10. Kistler AD, Serra AL. Novel diagnostic aspects of cystic renal diseases. [German]. *Nephrologe* 2010;5(5):375-83. |
| 11. PerSonalized Medicine - Genomic Phase. [Czech]. *Casopis lekaru ceskych* 2009;148(10):477-80. |
| 12. 太田充胤, 内田諭, 早川惠理, et al. PRES で発症し, 早期診断・手術に至った若年女性の副腎癌の 1 例. *日本内分泌学会雑誌* 2018;94 |
| 13. Pulciani S, Vittozzi A, S DI, et al. Rare diseases in post-genomic era. [Italian]. *Recenti Progressi in Medicina* 2017;108(7-8):307-15. |
| 14. Lesniak W. Rare diseases with epigenetic background. [Polish]. *Postepy biochemii* 2018;64(4):330-37. |
| 15. Li Y, Han Y, Cao P, et al. Research advances on medical genetics in China in 2015. [Chinese]. *Yi chuan = Hereditas / Zhongguo yi chuan xue hui bian ji* 2016;38(5):363-90. |
| 16. Du Y, Li Q. Research progress on the therapy of T-cell prolymphocytic leukemia. [Chinese]. *Chinese Journal of Clinical Oncology* 2017;44(21):1108-11. |
| 17. ゲノム・オミックス医療におけるビッグデータの利用. 日本臨床プロテオーム研究会要旨集 第 11 回日本臨床プロテオーム研究会; 2015. 日本臨床プロテオーム研究会. |
| 18. 郭茂祖, 武雪剑, 赵宁, et al. 一种基于多组学生物网络的癌症关键模块挖掘方法. *SCIENCE CHINA Life Sciences* 2014;57(57):951. |
| 19. 武志慧, 王飞, 姜召芸, et al. 健康医疗大数据与罕见病的精准用药. *科技导报* 2017;35(16):20-25. |
| 20. 孙丽雅, 张明, 贺林. “精准医学” 冷思考. *SCIENTIA SINICA Vitae* 2016;46(7):886-89. |
